# Co-targeting PIM and PI3K/mTOR using multikinase inhibitor AUM302 and a combination of AZD-1208 and BEZ235 in prostate cancer

**DOI:** 10.1038/s41598-020-71263-9

**Published:** 2020-09-01

**Authors:** Sabina Luszczak, Benjamin S. Simpson, Urszula Stopka-Farooqui, Vignesh Krishna Sathyadevan, Lina M. Carmona Echeverria, Christopher Kumar, Helena Costa, Aiman Haider, Alex Freeman, Charles Jameson, Marzena Ratynska, Imen Ben-Salha, Ashwin Sridhar, Greg Shaw, John D. Kelly, Hayley Pye, Kathy A. Gately, Hayley C. Whitaker, Susan Heavey

**Affiliations:** 1 Molecular Diagnostics and Therapeutics Group, University College London, London, UK; 2 Research Department of Pathology, University College London, London, UK; 3 Department of Uro-Oncology, UCLH NHS Foundation Trust, London, UK; 4 Trinity Translational Medicine Institute, St. James’s Hospital Dublin, Dublin 8, Ireland

**Keywords:** Cancer genomics, Cancer models, Cancer therapy, Urological cancer

## Abstract

PIM and PI3K/mTOR pathways are often dysregulated in prostate cancer, and may lead to decreased survival, increased metastasis and invasion. The pathways are heavily interconnected and act on a variety of common effectors that can lead to the development of resistance to drug inhibitors. Most current treatments exhibit issues with toxicity and resistance. We investigated the novel multikinase PIM/PI3K/mTOR inhibitor, AUM302, versus a combination of the PIM inhibitor, AZD-1208, and the PI3K/mTOR inhibitor BEZ235 (Dactolisib) to determine their impact on mRNA and phosphoprotein expression, as well as their functional efficacy. We have determined that around 20% of prostate cancer patients overexpress the direct targets of these drugs, and this cohort are more likely to have a high Gleason grade tumour (≥ Gleason 8). A co-targeted inhibition approach offered broader inhibition of genes and phosphoproteins in the PI3K/mTOR pathway, when compared to single kinase inhibition. The preclinical inhibitor AUM302, used at a lower dose, elicited a comparable or superior functional outcome compared with combined AZD-1208 + BEZ235, which have been investigated in clinical trials, and could help to reduce treatment toxicity in future trials. We believe that a co-targeting approach is a viable therapeutic strategy that should be developed further in pre-clinical studies.

## Introduction

Prostate cancer remains as the leading cause of cancer-related death for men^1^. Most current therapies exhibit issues with significant side effects, therefore it is crucial to develop lower toxicity therapeutics which would reduce the impact of treatment on patients’ lives.

Overexpression of the PIM family in prostate cancer has been found to lead to increased tumorigenicity and faster progression of the disease due to its impact on metastasis formation, invasion and migration^2–4^. Clinically, PIM can lead to decreased overall survival, insensitivity to cancer treatment and increased proliferation^5^. Its effect is mainly mediated by interactions with other pathways including PI3K/mTOR (Phosphoinositide 3-kinase; mammalian target of rapamycin), and various downstream effectors^2,6,7^.

The PI3K/mTOR pathway deregulation in cancer correlates with disease progression^8^ and impacts on apoptosis, survival and cell growth^6^. The PI3K pathway also regulates multiple oncogenes and tumour suppressor genes^8^. Despite being an attractive pathway for anti-cancer drug targeting, results from monotherapeutic PI3K inhibition strategies have been disappointing, with the growing consensus being that improved co-targeting strategies are warranted^9–11^.

The PIM and PI3K/mTOR pathways are interconnected, with each pathway influencing the signalling and activity of the other^12^. There is a significant overlap of cellular functions of PIM and AKT^6^. Moreover, both PIM and PI3K indirectly downregulate mTOR^6,13,14^. c-MYC is also upregulated by both PIM and mTOR^6^. This relationship gives rise to the development of resistance to treatment, as the pathways can bypass the inhibition by compensating for loss of signalling of either one^12,15,16^.

Early studies illustrated that combination treatments can have a synergistic effect on cell proliferation^17^, apoptosis, reduction of cell viability^18^ and cell growth^19^. AUM302, a novel triple PIM/PI3K/mTOR inhibitor, has recently been shown to increase cell differentiation, downregulate n-MYC, induce apoptosis and decrease cell viability in neuroblastoma^20^. Co-targeting of PIM and PI3K has been attempted in prostate cancer using different combinations of drugs^12,19^; these studies suggest that co-targeting PIM and PI3K could offer superior clinical outcomes to targeting either alone.

The proportion of prostate cancer patients that could benefit from the PIM-PI3K/mTOR pathway co-targeting is not well-understood or easy to estimate, as a wide range of alterations can result in abnormal pathway activation. The most commonly used biomarkers are PTEN deletion^21^ and PIK3CA mutation status^22^, however PTEN mutations are highly common in prostate cancer patients^22^ and they may not reflect the complex signalling regulation downstream from it^23^.

The aims of this investigation include identification of the potential benefit of the PIM-PI3K/mTOR co-targeted inhibition approach by analysis of publicly available data on prostate cancer patient populations. Moreover, we seek to determine the impact of co-targeted PIM and PI3K treatment on mRNA and phosphoprotein expression in prostate cancer cell models and ex vivo cultured prostate cancer tissue, as compared to targeting a single pathway.

## Results

### Around 20% of prostate cancer patients overexpress the targets of the drugs used in this study

**Figure 1:**
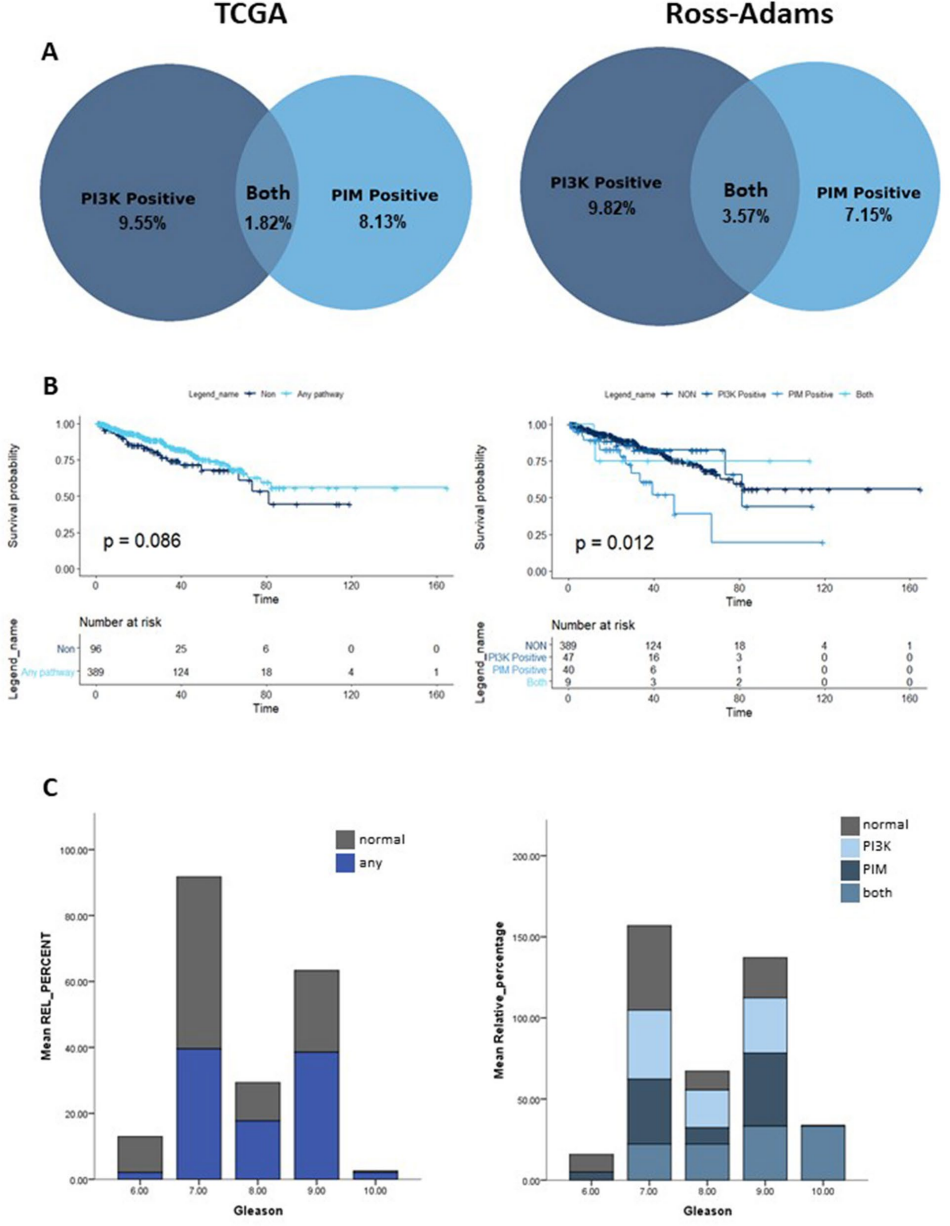
A high proportion of patients may be sensitive to PI3K/PIM inhibition. (**A**) Venn diagrams demonstrate the percentage of the prostate cancer cohort (TCGA or Ross-Adams, non-metastatic radical prostatectomy patients) that exhibited overexpression of the PI3K pathway, PIM pathway, or both. (**B**) Disease free survival probability of patients with any pathway upregulation versus no upregulation (left) and after separation into specific pathways (right). P-value was obtained using a Mantel-Cox test. (**C**) Distribution of Gleason grades within patient population groups. A higher Gleason score (1–5) indicates less well-differentiated prostate tissue and more aggressive disease. A Gleason grade is obtained by adding the Gleason scores of the two most prevalent tissue types in the sample. P-value was obtained using a Chi-square method.

In order to estimate the patient populations which could benefit from PI3K/PIM inhibition, publicly available genomic data were utilised. We hypothesised that an upregulation of the PI3K/mTOR or PIM pathways could make a patient more sensitive to PI3K or PIM treatment. PIM is regulated by transcription and is active when expressed^6^. mRNA expression can be an indicator of upregulation of other kinases, such as PI3K, which we hypothesize would result in sensitivity to treatment^7^. Patients were selected based on mRNA expression of the genes that are directly targeted by AZD-1208, BEZ235 and AUM302. Within the Ross-Adams dataset, 9.82% of patients overexpressed PIK3CA, PIK3CB, PIK3CG, PIK3CD or MTOR (termed PI3K positive), 7.14% overexpressed PIM1, PIM2 or PIM3 (termed PIM positive) and 3.57% of patients overexpressed at least one gene from both pathways. All patients who did not overexpress any of the target genes were termed ‘normal’. Similarly, in the TCGA cohort, 10.46% of patients were PI3K positive, 8.85% were PIM positive and 1.41% had overexpression in both pathways (Fig. 1A).

Based on our results patients overexpressing any genes directly targeted by these therapeutics represent between 20.53 and 20.72% of non-metastatic radical prostatectomy patients.

### PI3K and PIM pathway overexpressing patients exhibit more aggressive disease

We hypothesised that the patients identified as potentially sensitive to PIM/PI3K/mTOR inhibition would have signs of more aggressive disease. To this end, we looked at disease-free survival (DFS) and Gleason distribution between the identified groups.

In the TCGA cohort, the Kaplan–Meier curves revealed a non-statistically significant trend towards marginally reduced DFS in patients belonging to any of the identified groups compared to normal (p = 0.071). When separated into individual groups there was a statistically significant divergence of survival probabilities (p = 0.037) with PIM only positive patients showing reduced DFS (Fig. 1B).

We found that there was a significant increase in higher grade lesions whether comparing all groups to normal or in those who were PI3K positive, PIM positive or positive for both when compared to normal patients (Chi-squared p < 0.001, Fig. 1C). These differences were particularly contrasting in the case of Gleason grade 9 which made up only 25.3% of samples in the normal population, compared to 32.0%, 39.5% and 49.2% for PI3K positive only, PIM positive only and both pathway groups respectively (Supplementary Fig. S1, Supplementary Tables S1, S2, S3). These results indicate that the identified patient populations display a more aggressive disease.

### PIM family members are co-expressed with multiple genes of the PI3K/AKT/mTOR pathway

The PIM and PI3K pathways influence, and compensate, for each other in many ways. However, the extent, to which particular components of the signalling pathways are co-expressed in prostate cancer, is unclear. A better understanding of this relationship would allow us to predict which genes may be the most useful downstream targets to monitor efficacy following PIM or PI3K/mTOR inhibition, and which patient populations may be sensitive to the treatment. We looked at mRNA co-expression data of PIM and other genes in the PI3K pathway in the TCGA cohort. For the purpose of this investigation, Spearman’s correlation coefficient of 0.1–0.3 was described as a very weak correlation, 0.3–0.5 as weak and > 0.5 as moderate.

Using the TCGA cohort data we established that the three members of the PIM family are co-expressed at the mRNA level with different PI3K pathway genes at varying levels. Genes for this investigation were chosen to depict a wide range of well-studied targets downstream from PIM or PI3K pathways. Spearman’s correlation coefficient was used to determine the strength of the correlation of expression of PIM. PIM1, PIM2 and PIM3 all exhibit positive correlations, indicating they are both upregulated, with Bcl-6. Negative correlations, indicating one gene is downregulated while the other gene is upregulated, were seen between PIM genes and both p85α and mTOR. Overall, it would appear the pathways are predominantly mutually exclusive, particularly downstream, with some notable instances of co-expression (Figure S2).

### Triple kinase inhibition offers a superior inhibition of mRNA expression as compared to single kinase targeting

**Figure 2:**
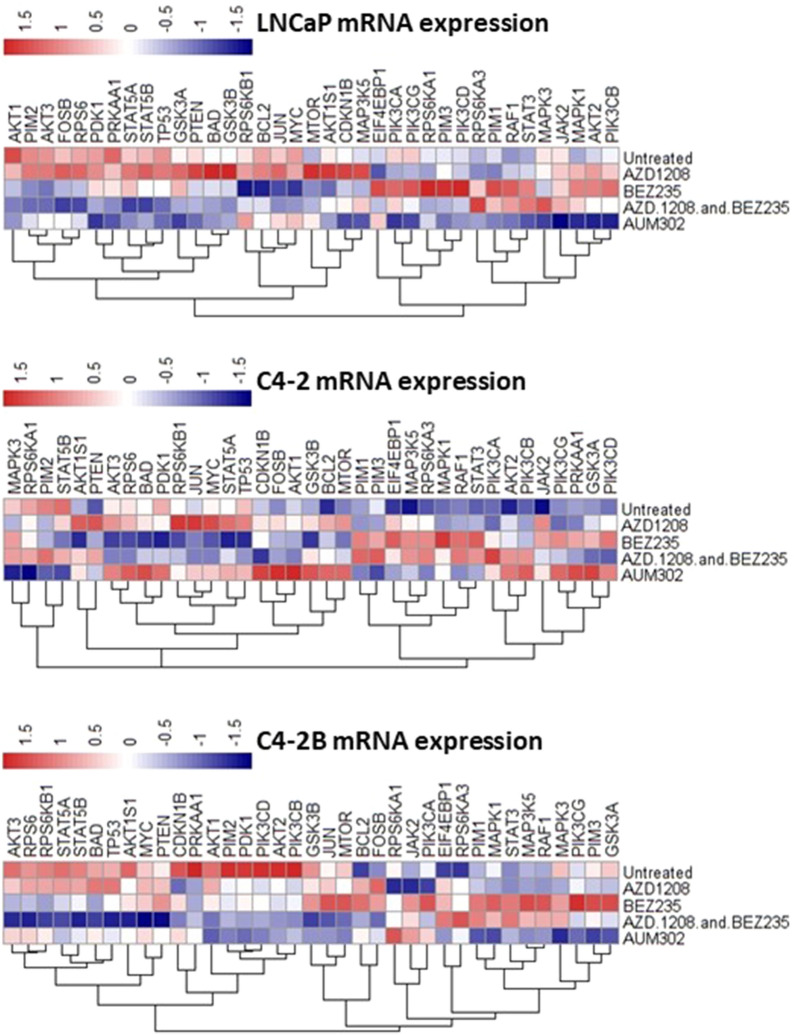
Co-targeting of PIM and PI3K/mTOR inhibits a wider range of genes than targeting either pathway alone. LNCaP, C4-2 and C4-2B cells were treated for 4 h with either AZD-1208 or BEZ235 alone, a combination of AZD-1208 and BEZ235, or the multikinase inhibitor AUM302. mRNA was extracted from the resulting samples and used for analysis of gene expression changes using Fluidigm. Results were then analysed using the ΔΔCt method and resulting relative quantification (RQ) values were plotted as a heatmap using R statistical environment version 3.3.1. Blue colour indicates downregulated expression, white no change, and red upregulated expression. One-way ANOVA with a Tukey post-test were used to determine the significance of the results, with significance for each test included in Supplementary Fig. S3.

Cell lines were used as in vitro prostate cancer models to determine changes in mRNA expression after treatment (Fig. 2). LNCaP, C4-2 and C4-2B cell lines were chosen as they represent increasingly aggressive forms of prostate cancer, having been isolated from one patient and then grown as invasive and ultimately metastatic xenografts. In some instances, AZD-1208 and BEZ235 caused opposite changes in mRNA expression, as upregulation following one of the treatments indicated a downregulation following the other, and vice versa e.g. PIM kinases, Raf1 or STAT3 in all cell lines.

Both AUM302 and a combination of AZD-1208 and BEZ235 inhibited a wider range of differentially expressed genes in the signalling pathways, as compared to AZD-1208 or BEZ235 alone. However, in the most aggressive cells, C4-2B, which represent bone metastases of prostate cancer, treatment with AUM302 or AZD-1208 + BEZ235 affected independent subsets of genes. The combination of AZD-1208 + BEZ235 inhibitors is more effective than AUM302 at inhibiting the genes upregulated by AZD-1208 alone e.g. AKT3, RPS6 or BAD (Fig. 2). A combination of AZD-1208 + BEZ-235 was also inferior to the multikinase AUM302 in inhibiting the genes upregulated by BEZ235 alone e.g. STAT3, MAPK1 or PIM1. This suggests that combination of AZD-1208 and BEZ235 is more effective against targets of BEZ235 than those of AZD-1208.

### PIM-PI3K/mTOR combined inhibition was more effective than monotherapy in reducing phosphoprotein levels, correlating with increased aggressivity of the cell line model used

**Figure 3:**
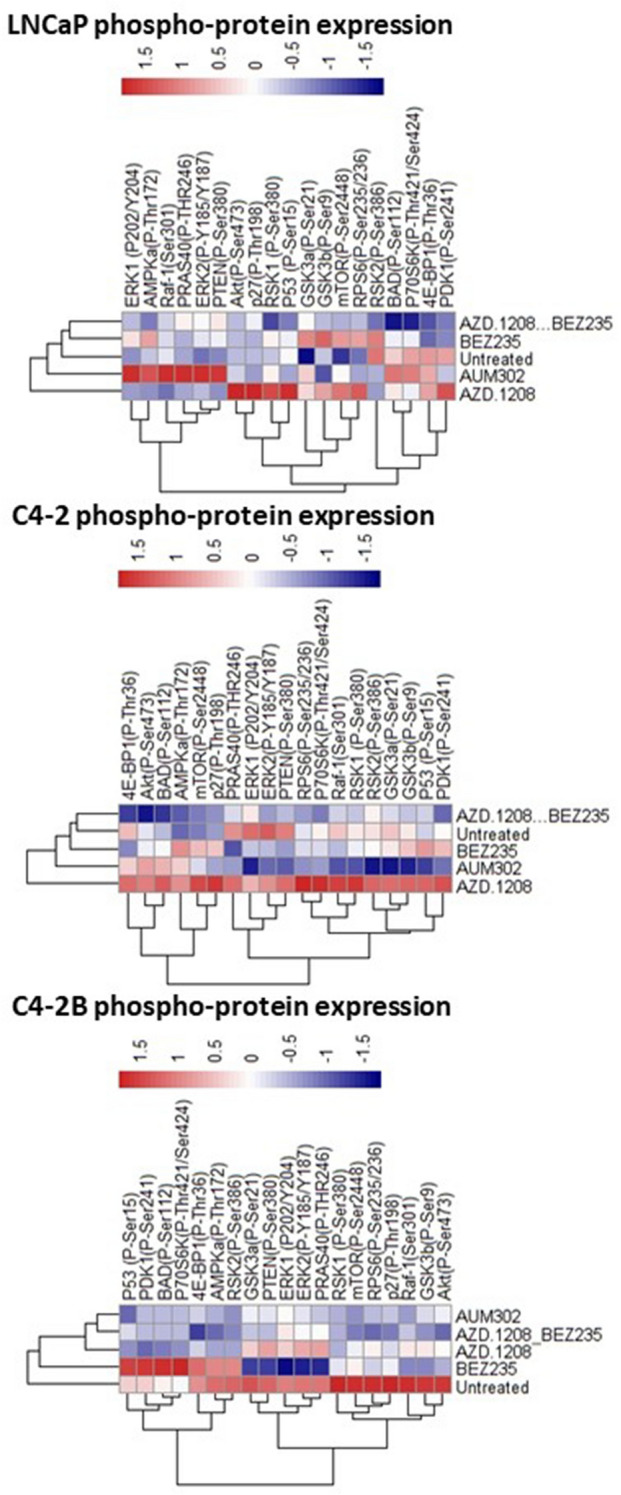
Inhibition of PIM and PI3K together using either a combination of AZD-1208 together with BEZ235, or AUM302 alone, showed superior inhibition of phosphoprotein levels than any of the monotherapies, correlating with increased aggressiveness. LNCaP, C4-2 and C4-2B cells were cultured and treated for 4 h with PIM inhibitor AZD-1208 or PI3K/mTOR inhibitor BEZ235 alone, a combination of AZD-1208 and BEZ235, or multikinase inhibitor AUM302, for four hours each. Proteins were then extracted and quantified using Bradford assay. Changes in phosphoprotein levels were quantified using a phosphokinase array which included key proteins of interest of the PI3K pathway. The results were normalised to positive and negative controls (a controlled amount of detection antibody or no antibody on the array), and then to the untreated control. One-way ANOVA and a Tukey post-test were used to determine the significance of the findings, included in full in Supplementary Figs. S5, S6 and S7. Heat-maps were generated using R statistical package version 3.3.1. Blue indicates downregulation of phosphoprotein expression, white no change, and red upregulation of expression.

Changes in phosphoprotein expression in cell line models post-drug treatment were assessed using a phosphokinase array (Fig. 3).

In LNCaP and C4-2 cells, treatment with AZD-1208 led to an upregulation of most of the phosphoproteins, such as AKT, mTOR or p27, which are part of either the PI3K/AKT/mTOR or the MAPK pathways, or downstream targets of either one. BEZ235 elicited a weak and often mixed effect in the same cell lines, e.g. downregulation of PRAS40, which is known to contribute to progression of prostate cancer^24^, upregulation of mTOR, and upregulation of phosphorylation of proteins such as GSK3a or GSK3b, which then lose their capability to negatively regulate proteins responsible for driving cell proliferation and survival^25^. In LNCaP cells, when AZD-1208 + BEZ235 were used in combination, it appears to be more effective than the multikinase inhibitor AUM302 at reducing phosphorylation of a wide range of substrates such as AMPKa, PRAS40 or BAD. In C4-2 cells, the efficacy of AUM302 and AZD-1208 + BEZ235 appears to be more consistent, with the multikinase inhibitor showing superior downregulation of phosphoprotein levels. The improved targeting of downstream targets by the co-targeting approaches seem to be most visible in the aggressive, metastatic cell line C4-2B.

### PI3K/mTOR-PIM combined inhibition inhibits proliferation in prostate cancer cell lines

**Figure 4:**
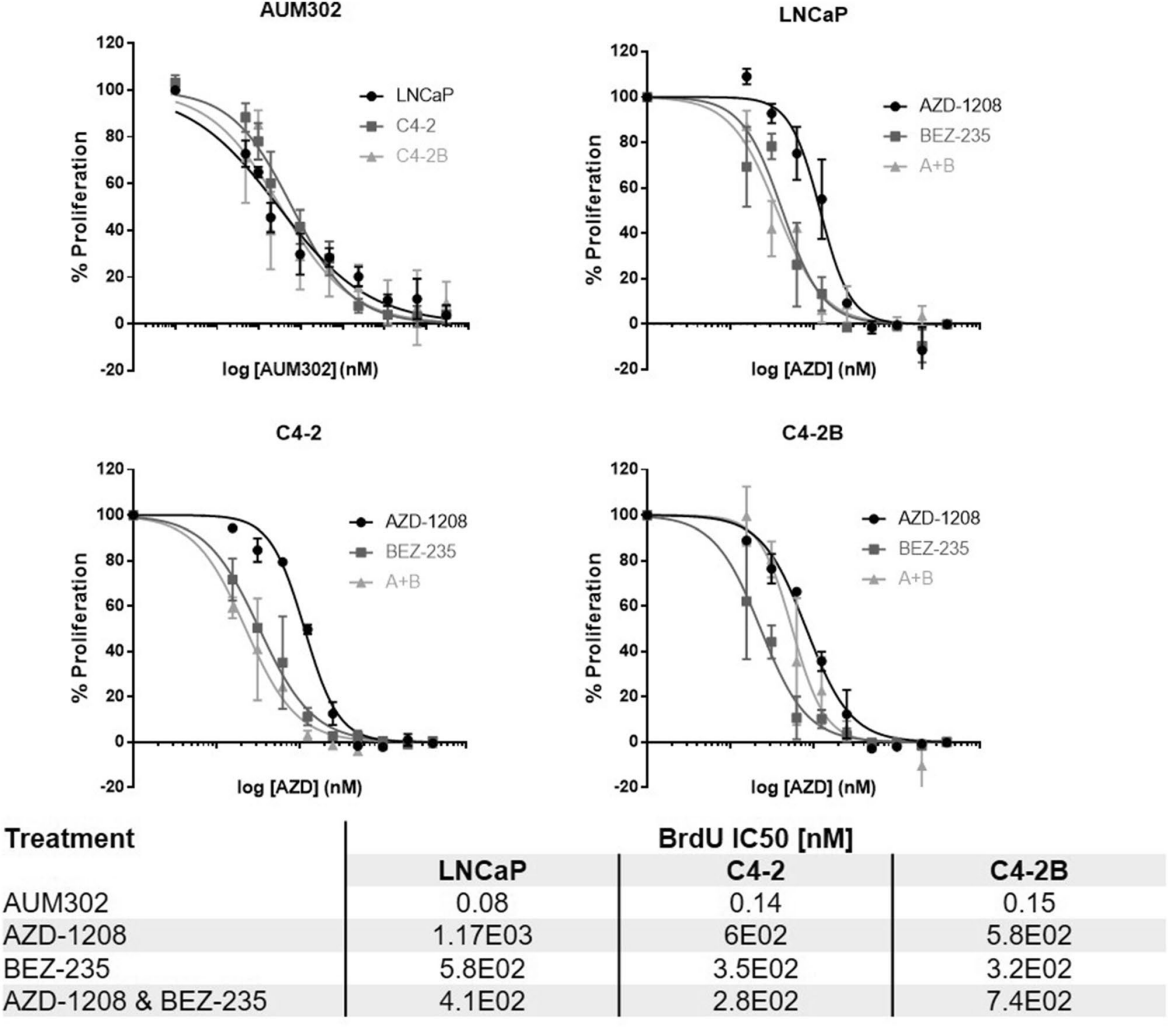
Co-targeted treatment approach was effective in reducing proliferation of prostate cancer cells. LNCaP, C4-2 and C4-2B cells were cultured and treated for 72 h using increasing concentrations of PIM inhibitor AZD-1208 or PI3K/mTOR inhibitor BEZ235 alone, a combination of AZD-1208 and BEZ235, or multikinase inhibitor AUM302. BrdU incorporation was performed using a colorimetric assay and used to assess the proliferation of the cells and to calculate IC50 values for each drug or drug combination.

LNCaP, C4-2 and C4-2B cell lines were treated with increasing doses of AZD-1208 or BEZ235 only, AZD-1208 and BEZ235 in combination or the multikinase inhibitor AUM302; proliferation was assessed via BrdU incorporation after 72 h. Treatment with either AZD-1208 or BEZ235 alone exhibited IC50s in the µM range, with the combination of both drugs leading to synergistic inhibitory effects at certain concentrations in the micromolar range (Supplementary Table S4)^26^. AUM302 treatment elicited dose-dependent inhibition of proliferation, with significantly lower IC50s of 0.08–0.15 nM (Fig. 4).

### Co-targeting PIM and PI3K in human prostate tumours ex vivo leads to anti-cancer effects

**Figure 5:**
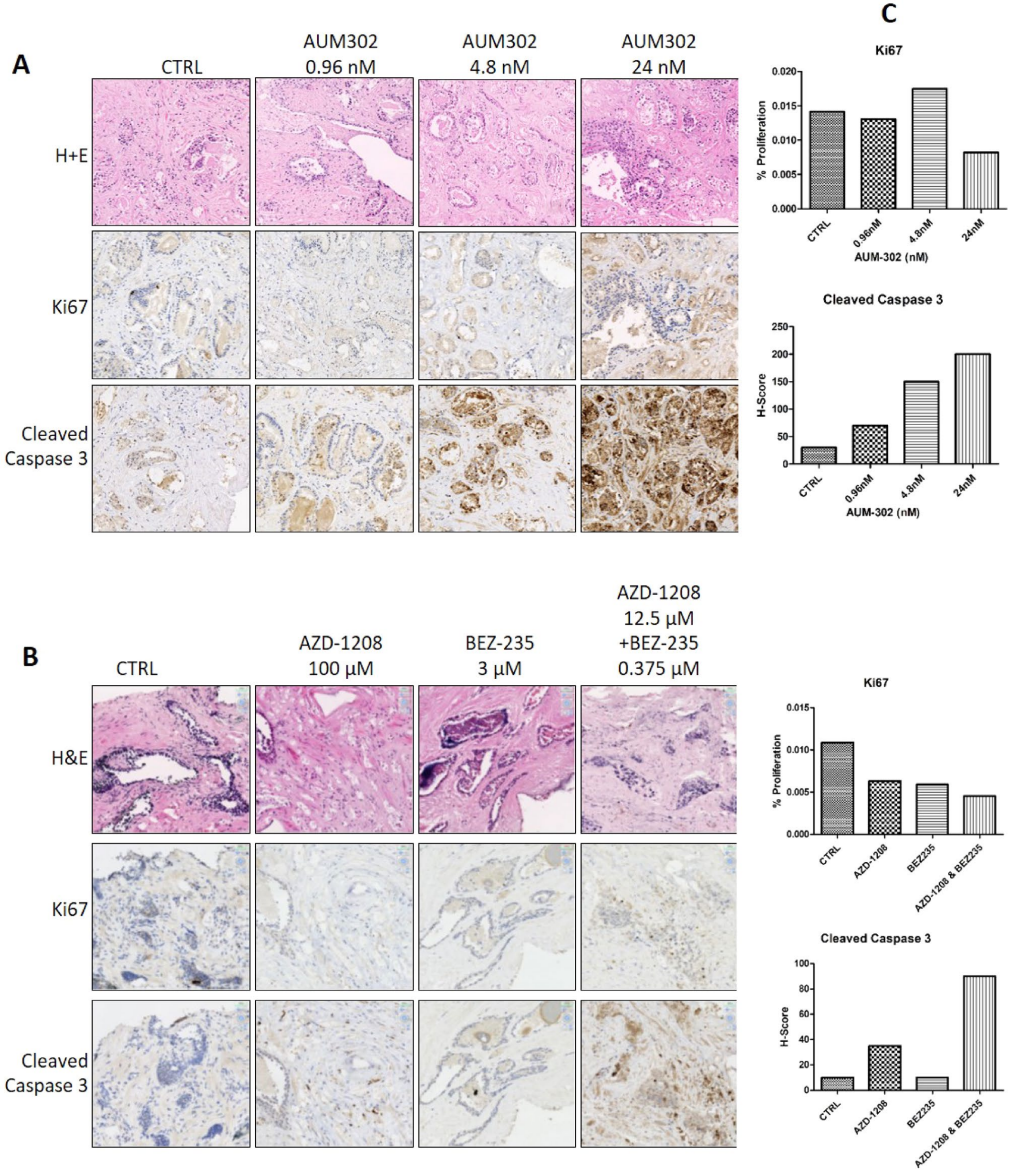
Morphology, proliferation and apoptosis of prostate cancer cells were significantly altered following treatment with AUM302 or a combination of AZD-1208 and BEZ235. (**A**) Human prostate tissue obtained from a biopsy was cultured for 72 h and treated with varying concentrations of AUM302 or (**B**) a combination of AZD-1208 and BEZ235. H&E staining, Ki67 and Cleaved caspase 3 were used to assess changes in morphology, proliferation and apoptosis, respectively. Brown staining indicates a positive antibody reaction, i.e. higher proliferation or apoptosis. Pink staining is specific for the cell cytoplasm, and blue for the nuclei. (**C**) Quantification was carried out using H scoring (cleaved caspase 3) and QuPath positive/negative nuclei count (Ki67).

Human prostate tissue was cultured ex vivo following Magnetic Resonance Imaging (MRI) guided tumour targeting^27,28^. Four patients were recruited for this study, all of whom underwent radical prostatectomy and had Gleason ≥ 3 + 4, ≥ Likert 3 disease (Supplementary Table S5). Tissue from patient PPL-0118 was treated with the multikinase inhibitor AUM302 at three concentrations, versus an untreated vehicle control. Morphology was altered, proliferation decreased and apoptosis increased with increasing doses of the drug (Fig. 5A). Tissue from patient PPL-0119 was treated with a combination of AZD-1208 and BEZ235, at low, medium and high doses. Here images are shown from high doses of AZD-1208 and BEZ235 separately, and with a low dose combination of AZD-1208 and BEZ235 (Fig. 5B). The low dose combination in particular led to dramatic alterations in morphology, proliferation and apoptosis in comparison with treatment with either inhibitor alone, or untreated cells (Fig. 5B). Quantification of images from each patient was carried out using H scoring (cleaved caspase 3) and positive/negative nuclei counts (QuPath, Ki67) Fig. 5C).

## Discussion

PIM and PI3K pathways have been shown to interact and influence the progression and development of several cancers, including prostate^2,8^. Here, for the first time in prostate cancer, we investigated the efficacy of a novel multikinase PIM/PI3K/mTOR inhibitor AUM302 and a combination of AZD-1208 and BEZ235, well-researched inhibitors of PIM and PI3K/mTOR, respectively.

Our initial investigation into the population that may benefit from the co-targeted therapy approach revealed that a large subset of prostate cancer patients overexpress the direct targets of the drugs we studied (ca. 20%), and thus they may benefit from a triple kinase inhibition approach. This population may also have more aggressive disease. Our data adds to the growing understanding of the role of PIM in the pathogenesis of prostate cancer^2,3,5,29^. As PIM may be responsible for driving resistance to PI3K inhibitors^5,23^, the use of a co-targeted strategy may benefit not only patients that are both PI3K and PIM positive, but also those with upregulation of genes in a single pathway. Currently, targeted therapeutics inhibit pathways dysregulated at a similar level; perhaps the most notable example being trastuzumab for HER2 positive breast cancer (upregulated in ∼ 20–30% cases)^30–32^. Therefore, the development of an effective therapy against PIM and PI3K could be clinically important, as it would benefit a significant proportion of prostate cancer patients.

A potential limitation of our study is the method used to identify patients who may be sensitive to PI3K/PIM inhibition. However, we believe that our findings most likely underestimate the true population due to the high expression cut-off used for selection, and exclusion of other common PI3K pathway activators such as PTEN loss.

The relationship between PIM and PI3K signalling has been well-described, as PIM can imitate the effects of AKT, influence mTOR and stimulate its downstream targets^6,13,14^. Our data appears to support this relationship, as it would explain the upregulation of mTOR phosphoprotein following treatment with a PIM inhibitor, or upregulation of PIM targets such as BAD phosphoprotein after treatment with BEZ235. This pathway compensation has been shown to contribute to development of resistance to PI3K inhibitors^5,33,34^. We noted that the effects of AZD-1208 and BEZ235 on gene and phosphoprotein expression often oppose each other. A wide range of genes are upregulated following treatment with AZD-1208, while being downregulated by BEZ235 or vice versa. We hypothesise that this can be attributed to the existence of the aforementioned compensatory mechanisms.

AUM302 and the combination of AZD-1208 + BEZ235 affect a different set of genes and phosphoproteins, despite theoretically targeting the same proteins. AUM302 seems to affect similar genes to AZD-1208, which might suggest it preferentially targets the PIM pathway, despite exhibiting a lower IC50 for PIK3CA^20^. The combination of AZD-1208 + BEZ235, appears to exhibit greater inhibitory effects towards PI3K/mTOR. Phosphoprotein expression suggests this disparity becomes less significant in more aggressive cell lines, where the combined inhibition approach is particularly effective. This supports previous work in AML that suggested there is a relationship between PIM expression and the cell’s response to PIM inhibition^35^.

AUM302 elicited significant responses both in vitro and ex vivo, with low IC50s in the nM range. AZD-1208 and BEZ235 were less effective, however, they did elicit a significant response in the one patient tumour, where a much lower combined dose led to obvious morphological changes and cell death in comparison with high doses of either drug alone. The strategy of co-targeting signalling pathways separately validates previous studies^19^ which noted synergistic inhibition in prostate models treated with a combination of PIM and PI3K or AKT inhibitors.

While the combination of AZD-1208 and BEZ235 did lead to synergistic responses in vitro, these were at concentrations that are unlikely to lead to clinical benefit. The significantly lower IC50 of AUM302, as compared to AZD-1208 and BEZ235 in combination, could suggest a clinical benefit of this novel inhibitor in future work. Previously, PI3K pathway inhibitors were not as successful as first hoped in clinical trials, often due to innate or acquired treatment resistance and excessive dose-limiting toxicity^15,36^. With an IC50 an order of magnitude lower than the AZD-1208 + BEZ235 combination, the multikinase inhibitor could potentially reduce adverse effects and allow for the co-targeting approach to be used clinically. Mohlin et al. also reported the IC50 for AUM302 in neuroblastoma models to be in the nanomolar range^20^. Furthermore, adverse effects are a major issue of non-targeted prostate cancer treatments^37^; the development of new therapeutic strategies that would have less impact on the patients’ quality of life could significantly improve the current standard of care. As we have demonstrated the comparable molecular effects of the AUM302 as compared to the combined agents AZD-1208 + BEZ235, we believe that it should be brought forward to further preclinical testing and ultimately clinical trials, especially given that AZD-1208 and BEZ235 have separately been brought to trials previously.

## Conclusions

Our investigation supports the emerging concept of co-targeting PIM and PI3K as a valid therapeutic approach in prostate cancer. Analysis of gene and phosphoprotein expression following treatment with AZD-1208, BEZ235, a combination of AZD-1208 and BEZ235, and AUM302 suggests that monotreatment often results in upregulation of off-target genes and proteins, possibly due to the existence of a compensatory feedback between PIM and PI3K pathways. AUM302 and AZD-1208 + BEZ235 were more successful at inhibiting a wide range of targets, suggesting that it may be a valid approach to tackling resistance to PI3K treatment. Moreover, the multikinase inhibitor AUM302 offered superior functional effects regarding suppression of proliferation over AZD-1208 + BEZ235 and had a significantly lower IC50, which could potentially indicate it could reduce dose-dependent treatment toxicity and should be further investigated as a co-targeted treatment strategy for PIM and PI3K. This work supports the growing evidence that PIM kinases may be best targeted in cancer using a combined inhibition approach, alongside other pathways including PI3K.

## Materials and methods

### Publicly available patient populations

#### TCGA Prostate adenocarcinoma (PRAD) provisional cohort

In brief, as part of The Cancer Genome Atlas (TCGA), data from 499 surgically resected, untreated, primary prostate carcinomas was downloaded from The Cancer Genome Atlas Broad Firehouse portal (available: https://gdac.broadinstitute.org/)^38^.

#### Ross-Adams prostate cancer cohort

Previously published background normalised microarray expression data was retrieved from the National Centre for Biotechnology Information gene expression omnibus (NCBI GEO) database^39^. mRNA was extracted from primary prostate cancer resected from radical prostatectomy without prior treatment. Bead level data were pre-processed to remove spatial artefacts.

### Estimation of drug-sensitive populations

RNAseq data was mean-centred and converted to relative z scores using the R statistical environment (version 3.3.1). Patients were subdivided based on overexpression (z score greater than 2) of one or more members of the PI3K pathway targeted by AZD-1208, BEZ235 and AUM302 namely: PIK3CA, PIK3CB, PIK3CG, PIK3CD and MTOR (PI3K positive) or overexpressing PIMs 1–3 (PIM positive). Patients not meeting this expression level and within 2 standard deviations from the population mean were termed ‘normal’, those appearing in both populations were also noted.

Kaplan–Meier estimator curves from the derived populations in the TCGA were produced using disease-free survival data (merged clinical level 1 data).

### Cell culture

LNCaP, C4-2 and C4-2B cell lines, representative of increasingly aggressive prostate cancer, were obtained from ATCC and cultured using Rosewell Park Memorial Institute (RPMI) 1640 media (Gibco) with 10% foetal bovine serum (Gibco). LNCaP cells are human prostate cancer cells derived from a lymph node; C4-2 cells were obtained by inoculation of LNCaP cells subcutaneously into a castrated mouse; C4-2B cells followed a subsequent inoculation of C4-2 cells with osteosarcoma fibroblasts. Cells were routinely tested for mycoplasma contamination prior to treatments (LookOut Mycoplasma PCR Detection Kit from Sigma Aldrich).

### PIM and PI3K pathway inhibition

LNCaP, C4-2 and C4-2B cells were treated with AZD-1208 (PIM inhibitor; Astrazeneca), BEZ235 (also known as Dactolisib, PI3K/mTOR inhibitor; Novartis), AUM302 (previously known as IBL-302, PIM/PI3K/mTOR inhibitor; AUM Biosciences. AUM-302 was previously IBL-302 compound licensed by Inflection Biosciences from The Spanish National Cancer Centre CNIO), or a combination of AZD-1208 and BEZ235. The vehicle for all drugs was the RPMI 1640 media with 10% FBS. Drug concentrations were previously optimised for the purpose of these experiments and are as follows: AZD-1208—6.25 μM; BEZ235—0.293 µM; AUM302—0.16 nM. Treatment duration was 4 h, as optimized using quantitative PCR (Supplementary Fig. S3).

### RNA extraction and cDNA synthesis

RNA extraction was performed using the RNeasy Mini Kit from Qiagen according to a previously optimized protocol. cDNA was synthesized using the High Capacity cDNA Reverse Transcription Kit from Applied Biosystems.

### Fluidigm

Real-time PCR technology using Fluidigm was employed to investigate changes in gene expression following drug treatments. Primers were chosen to reflect key interactions of the PI3K and PIM pathways, including MAPK3, RPS6KA1, PIM2, STAT5B, AKT1S1, PTEN, AKT3, RPS6, BAD, PDK1, RPS6KB1, JUN, MYC, STAT5A, TP53, CDKN1B, FOSB, AKT1, GSK3B, BCL2, MTOR, PIM1, PIM3, EIF4EBP1, MAP3K5, RPS6KA3, MAPK1, RAF1, STAT3, PIK3CA, AKT2, PIK3CB, JAK2, PIK2CG, JAK2, PIK3CG, PRKAA1, GSK3A, PIK3CD. Samples were tested in biological triplicates and primers in technical triplicates. Data was normalised using the ΔΔCt method^40^. One-way ANOVA with Tukey post-test were used to determine statistical significance of mRNA expression.

### Phosphokinase arrays

Proteins were isolated using Radioimmunoprecipitation assay buffer (RIPA buffer) supplemented with protease and phosphatase inhibitor cocktails from the C-Series phosphorylation array C1 (RayBio). Bradford assay was performed as described^41^ to quantify the amount of total protein and normalize their concentration. Phosphokinase array kits were obtained from RayBio (Human/Mouse AKT Pathway Phosphorylation Array C1) to represent key proteins of interest in the relevant pathways including ERK1, ERK2, PRAS40, GSK3A, PTEN, GSK3B, RAF-1, mTOR, RPS6, Akt, p27, RSK1, AMPKa, p53, RSK2, BAD, P70S6K, 4E-BP1 and PDK1. The arrays were performed according to the protocol, except ten washes were performed at each wash step instead of five. Images were developed on X-ray films using a Konica SRX101A Processor. Phosphokinase array images were quantified using ImageJ 1.52a by measuring pixel intensity and the normalising the values to both positive and negative controls. One-way ANOVA with Tukey post-test were used to determine statistical significance of phosphoprotein expression.

### Ex vivo culture, immunohistochemistry and staining

Matched tumour and benign human prostate tissue was collected from patients who had provided informed consent under UCL/UCLH Biobank ethics (REC 15/YH/0311), and sampled as per the PEOPLE method^27,42^. Tissue was cultured ex vivo and treated with AZD-1208, BEZ235, a combination of AZD-1208 and BEZ235, AUM302 or untreated, using the gelatin sponge method for 72 h^27^. Samples were used to create a tissue microarray and staining with H + E to assess morphology, as well as immunohistochemistry using Leica Biosystems BOND-MAX Automated IHC/ISH Stainer using antibodies Ki67 (Dako, M7240, 1:100, ER2 20 min), quantified for percentage positive nuclei using QuPath and cleaved Caspase 3 (Cell Signaling, 9664, 1:200, ER2 20 min), quantified by H-Score.

### BrdU index

Cell proliferation was assessed using a colorimetric assay of BrdU incorporation (Roche). LNCaP, C4-2 and C4-2B cell lines were treated with AZD-1208, BEZ235, a combination of AZD-1208 and BEZ235, and AUM302 for 72 h at increasing drug concentrations in the micromolar range for AZD-1208 and BEZ235, and nanomolar range for AUM302, and assayed as per manufacturer’s protocol. The resulting data was used to determine cell line specific IC50s via non-linear regression using GraphPad Prizm 7.

### PIM family co-expression

Co-expression of PIM1, PIM2 and PIM3 mRNA with the PI3K pathway was determined using the TCGA cohort, as described above. Spearman’s correlation coefficient (Spearman’s R) was used to quantify the relationship, with significance level obtained from a two-sided t-test.

### Statistical analysis and data processing

All statistical analyses of publicly available datasets were conducted using IBM SPSS statistical analysis software (for Windows version 22.0) and visualised using R version 3.3.1 (packages: Dplyr and ggplot)^43^. Chi-squared tests were used to compare frequency counts of alterations between clinical groups, for all Kaplan–Meier plots/survival analysis, univariate analysis were carried out using Log-Rank (Mantel-Cox) tests. Kaplan–Meier curves were constructed and plotted using the survminer and survival r packages. Proportional Venn diagrams were created using the eulerr R package^44^.

Statistical analyses of experimental data were performed with GraphPad Prism version 7 or R statistical environment version 3.3.1.

### Ethics statement

All experimental protocols were approved by UCLH/UCLH Biobank Research Ethics Committee (REC 15/YH/0311), all patients recruited for this study gave informed consent under these ethics, and all methods were carried out in accordance with relevant guidelines and regulations.

## Supplementary information

Supplementary information.
