# Author Correction: Co-targeting PIM and PI3K/mTOR using multikinase inhibitor AUM302 and a combination of AZD-1208 and BEZ235 in prostate cancer

**DOI:** 10.1038/s41598-020-76679-x

**Published:** 2020-11-11

**Authors:** Sabina Luszczak, Benjamin S. Simpson, Urszula Stopka-Farooqui, Vignesh Krishna Sathyadevan, Lina M. Carmona Echeverria, Christopher Kumar, Helena Costa, Aiman Haider, Alex Freeman, Charles Jameson, Marzena Ratynska, Imen Ben-Salha, Ashwin Sridhar, Greg Shaw, John D. Kelly, Hayley Pye, Kathy A. Gately, Hayley C. Whitaker, Susan Heavey

**Affiliations:** 1 Molecular Diagnostics and Therapeutics Group, University College London, London, UK; 2 2Research Department of Pathology, University College London, London, UK; 3 Department of Uro‑Oncology, UCLH NHS Foundation Trust, London, UK; 4 Trinity Translational Medicine Institute, St. James’s Hospital Dublin, Dublin 8, Ireland

Correction to: *Scientific Reports* 10.1038/s41598-020-71263-9, published online 1 September 2020

In this Article, Figure 1 and its accompanying legend are incorrect. The correct Figure 1 and legend appear below.

**Figure 1:**
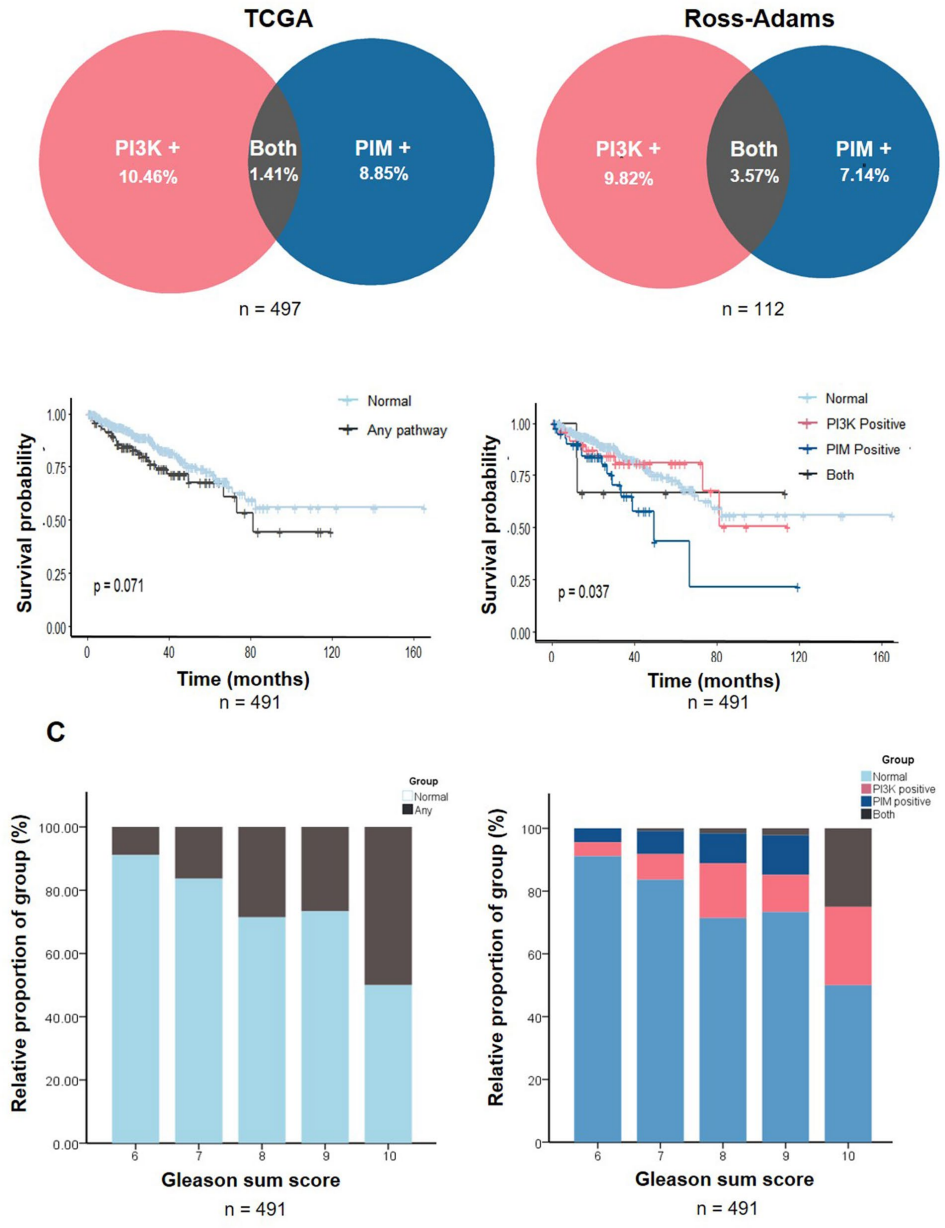
A correct version of the original Figure 1. A high proportion of patients may be sensitive to PI3K/PIM inhibition. (**A**) Venn diagrams demonstrate the percentage of the prostate cancer cohort (TCGA or Ross-Adams, non-metastatic radical prostatectomy patients) that exhibited overexpression of the PI3K pathway, PIM pathway, or both. (**B**) Disease free survival probability of patients with any pathway upregulation versus no upregulation (left) and after separation into specific pathways (right). P-value was obtained using a Mantel-Cox test. (**C**) Distribution of Gleason grades within patient population groups. A higher Gleason score (1–5) indicates less well-differentiated prostate tissue and more aggressive disease. A Gleason grade is obtained by adding the Gleason scores of the two most prevalent tissue types in the sample. P-value was obtained using a Chi-square method, and is plotted as a proportion of the patients within each Gleason sum group that fall into each expression group (PIM positive, PI3K positive, both positive or any positive), with colours corresponding to parts A and B.

