# Supplementary material for: Co-targeting PIM and PI3K/mTOR using multikinase inhibitor AUM302 and a combination of AZD-1208 and BEZ235 in prostate cancer
Source: Sci Rep. 2020 Sep 1;10:14380. doi: 10.1038/s41598-020-71263-9 (PMC7463239; doi:10.1038/s41598-020-71263-9)
Supplement: Supplementary file 1 — Supplementary information. [file 41598_2020_71263_MOESM1_ESM.docx]

SUPPLEMNTARY MATERIALS FILE

Co-targeting PIM and PI3K/mTOR using multikinase inhibitor AUM302 and a combination of AZD-1208 and BEZ235 in prostate cancer

Sabina Luszczak^1^, Benjamin S Simpson^1^, Urszula Stopka-Farooqui^1^, Vignesh Krishna Sathyadevan^1^, Lina M Carmona Echeverria^1^, Christopher Kumar^1^, Helena Costa^2^, Aiman Haider^2^, Alex Freeman^2^, Charles Jameson^2^, Marzena Ratynska^2^, Imen Ben-Salha^2^, Ashwin Sridhar^3^, Greg Shaw^3^, John D Kelly^3^, Hayley Pye^1^, Kathy A Gately^4^, Hayley C Whitaker^1†^, Susan Heavey^1†*^.

^1^Molecular Diagnostics and Therapeutics Group, University College London, London, UK

^2^Research Department of Pathology University College London, London, UK

^3^Department of Uro-Oncology, UCLH NHS Foundation Trust, London, UK

^4^Trinity Translational Medicine Institute, St. James's Hospital Dublin, Dublin 8, Ireland

^†^ These authors contributed equally to the work

***** Correspondence: Susan Heavey, s.heavey@ucl.ac.uk


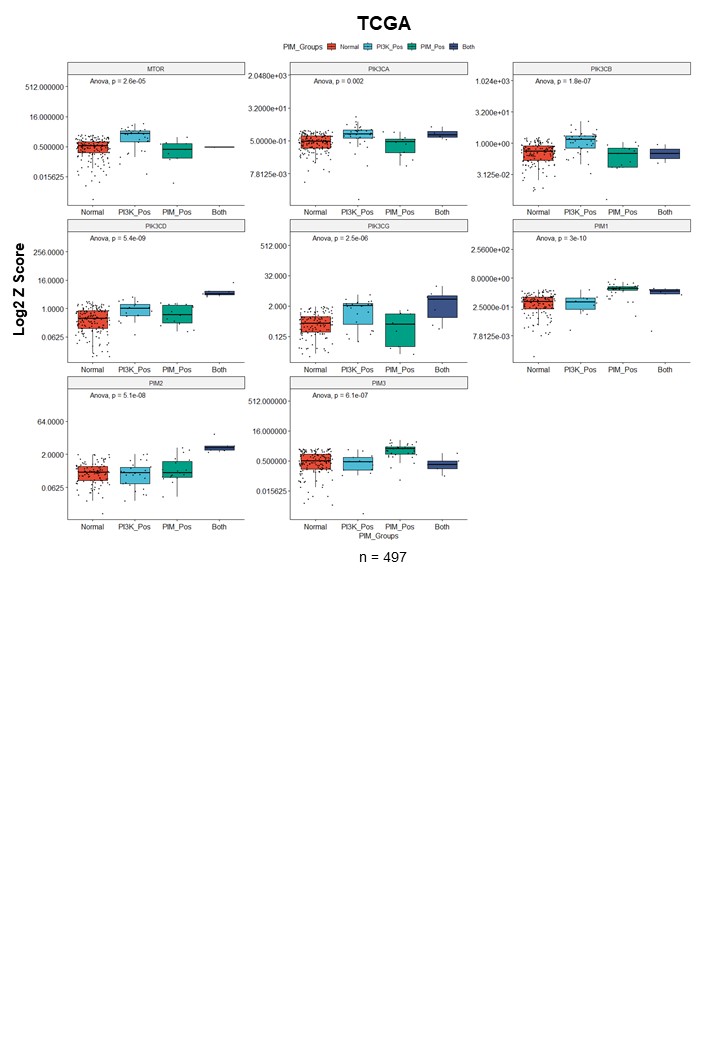


**Figure S1** Expression of constituent genes across derived groups. Key genes targeted by selected therapeutics were used to estimate the potentially sensitive patient population using mRNA as a surrogate for increased activity. The above panel shows all constituent gene mRNA expression (as normalized z scores) across PI3K positive, PIM positive, PI3K+PIM positive or normal (PIM and PI3K negative) patient groups in the TCGA cohort (obtained from the broad Firehose). Boxplots represent the mean z-score, interquartile range and the range of the data. Genes of interest include: PI3KCA, PI3KCB, PI3KCG, PI3KCD, MTOR, PIM1, PIM2, and PIM3. Statistical comparisons are shown in Table S1 below.


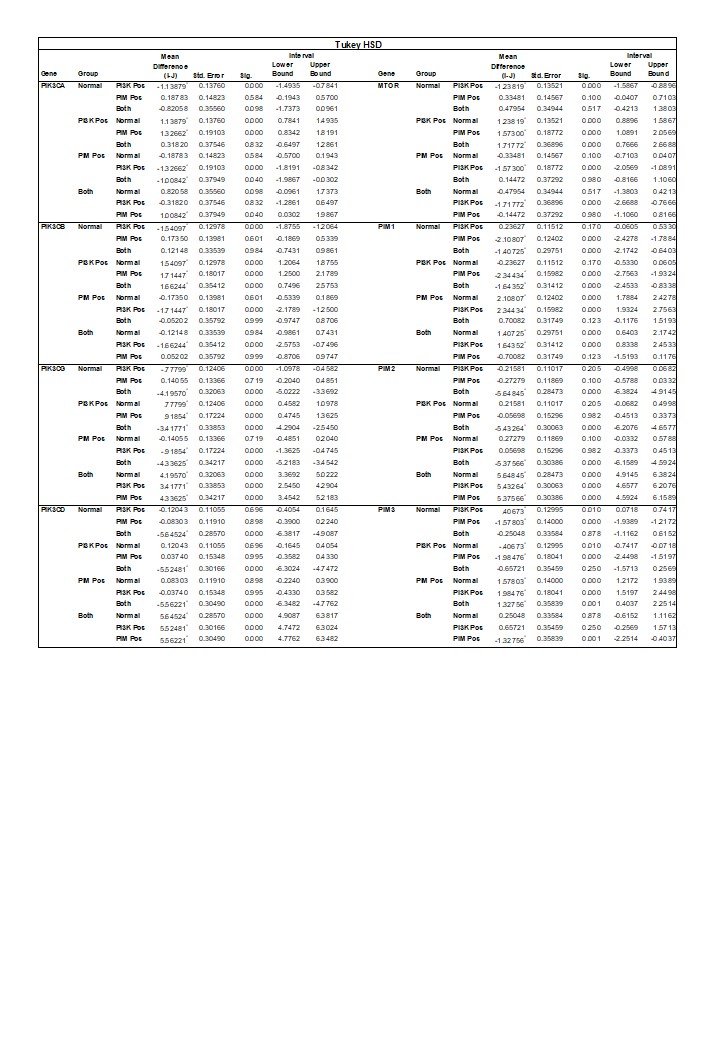


**Table S1** Statistical results of expression of constituent genes across derived groups from Figure S1.1. Gene expression was compared between derived patient groups – PIM positive, PI3K positive, PIM and PI3K positive and normal using a one-way ANOVA (p = <0.05) followed by a Tukey HSD post-hoc test. Table S1 shows the dependent variable (expression of gene of interest) between the derived groups. Columns indicate p value and 95% confidence intervals respectively. An alpha value of p = <0.05 was considered statistically significant.


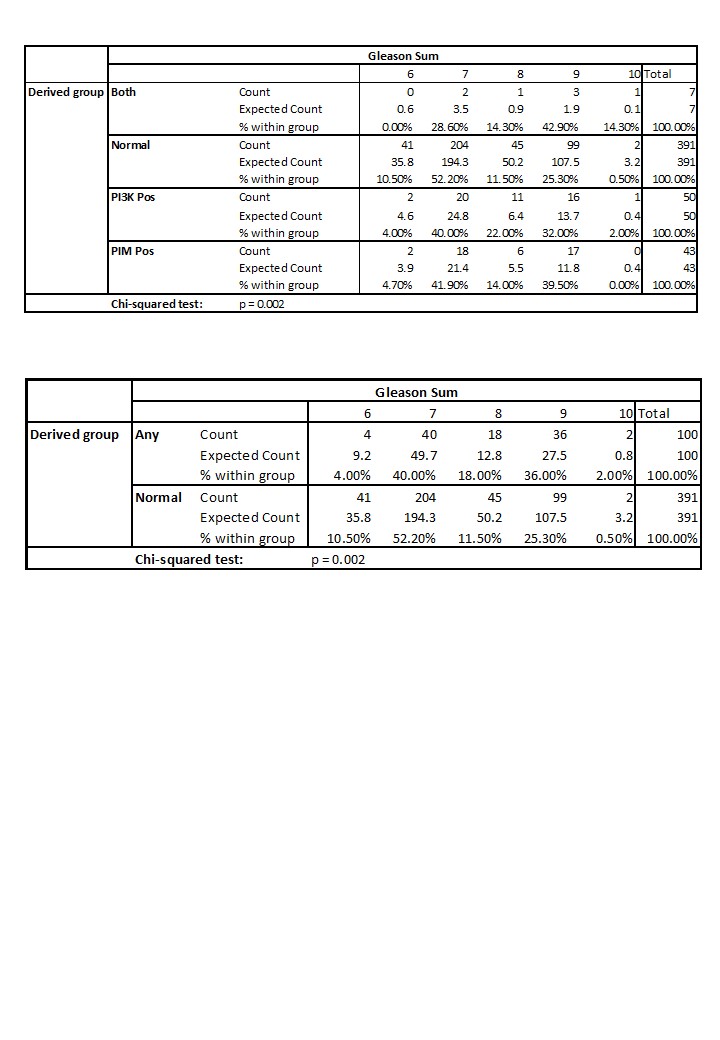


**Table S2** Cross-tabulation from the TCGA cohort representing the numbers (count) of patients that are PIM positive, PI3K only positive, PIM and PI3K positive, or normal (not positive for PIM or PI3K), broken down into Gleason sum categories (data displayed in Figure 1C). Rows display the patient count per group, the probabilistic expected count and the representation of that Gleason sum within the derived patient group. The results of the Pearson Chi-Square test investigating the relationship between the expected distribution of Gleason sum between derived patient groups and the actual distribution of Gleason sum between groups is also shown (asymptotic 2-sided p-value).


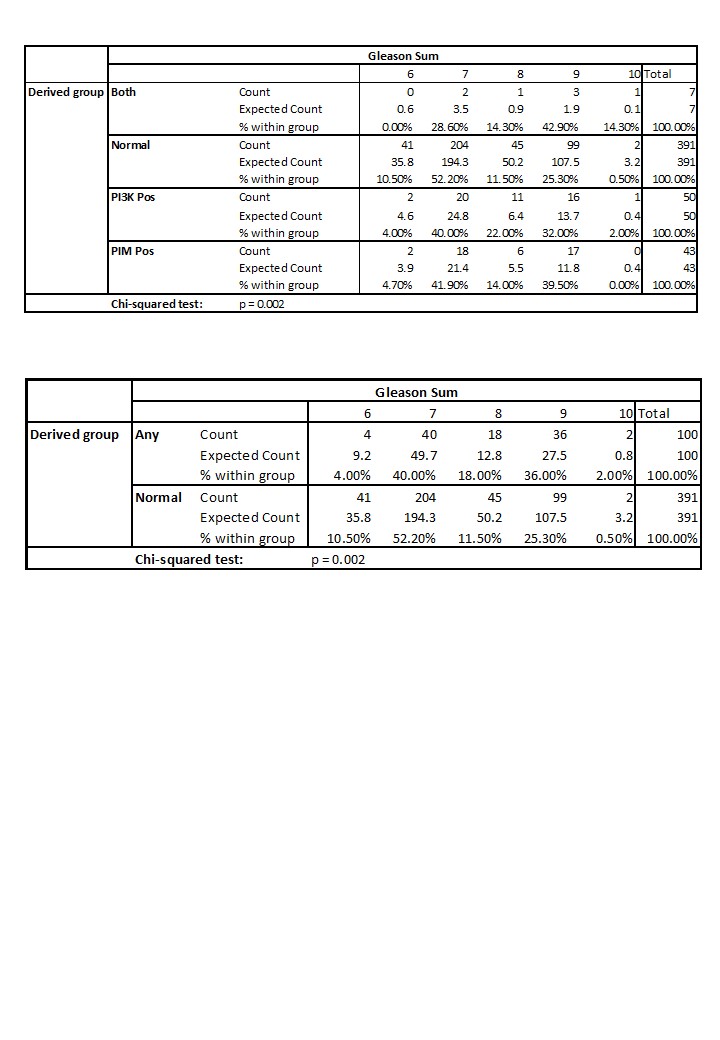


**Table S3a** Cross-tabulation from the TCGA cohort representing the numbers (count) of patients that are normal (not positive for PIM or PI3K) or those whom were PI3K, PIM or positive for both pathways (any) broken down into Gleason sum categories (data displayed in Figure 1C). Rows display the patient count per group, the probabilistic expected count and the representation of that Gleason sum within the derived patient group. Key: 1 represents normal, 2 is PI3K positive, PIM positive or both PIM and PI3K positive. The results of the Pearson Chi-Square test investigating the relationship between the expected distribution of Gleason sum between derived patient groups and the actual distribution of Gleason sum between groups is also shown (asymptotic 2-sided p-value).

Our method gave equal weights to each gene within the panel, allowing us to analyse the mean gene expression within the signalling pathways across the different groups (Figure S1, Table S1, Table S2, Table S3). Expression of PIK3CA, PIK3CB, PIK3CG and MTOR were all significantly higher in the PI3K positive group compared to normal with the exception of PIK3CD which was only raised in patients who were positive for both pathways (Figure S1, Table S1, Table S2, Table S3). Likewise, expression of all three PIM genes was significantly higher in the PIM only positive group (Figure S1, Table S1, Table S2, Table S3). Patients who were positive in both pathways had significantly higher PIK3CG, PIK3CD and PIM2 expression compared to all others (Figure S1, Table S1, Table S2, Table S3).

| **Group** | **Both** | **Normal** | **PI3K Positive** |
| --- | --- | --- | --- |
| **Normal** | 0.873 | - | - |
| **PI3K Positive** | 0.873 | 0.906 | - |
| **PIM Positive** | 0.906 | 0.021 | 0.185 |

**Supplementary table S3b.** Post-hoc pairwise comparisons of disease-free survival for determined patient groupings based on mRNA pathway overexpression. Values shown indicate the resulting p-values following pairwise Log-Rank testing followed by Benjamini-Hochberg correction.

**
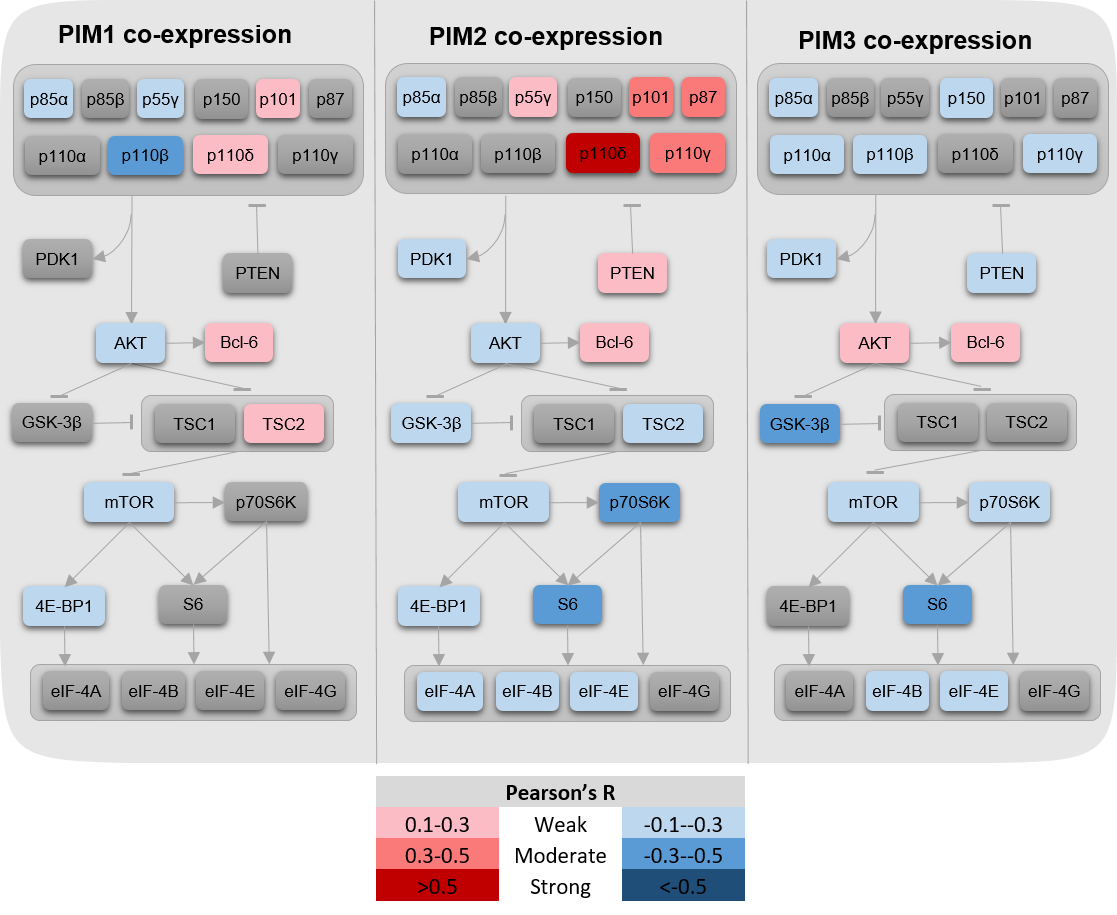
**

**Figure S2.** PIM family co-expression with the PI3K/AKT/mTOR pathway. The pathway diagrams were constructed after analysis of the TCGA cohort and Spearman’s correlation coefficient was used to identify the extent of the co-expression. Significance level was determined using a two-sided t-test. Spearman’s correlation coefficient: 0.1-0.3 very weak, 0.3-0.5 weak, >0.5 moderate.

*PIM family members are co-expressed with multiple genes of the PI3K/AKT/mTOR pathway*

The PIM and PI3K pathways influence, and compensate, for each other in many ways. However, the extent, to which particular components of the signalling pathways are co-expressed in prostate cancer, is unclear. A better understanding of this relationship would allow us to predict which genes may be the most useful downstream targets to monitor efficacy following PIM or PI3K/mTOR inhibition, and which patient populations may be sensitive to the treatment. We looked at mRNA co-expression data of PIM and other genes in the PI3K pathway in the TCGA cohort. For the purpose of this investigation, Spearman’s correlation coefficient of 0.1-0.3 was described as a very weak correlation, 0.3-0.5 as weak and >0.5 as moderate.

Using the TCGA cohort data we established that the three members of the PIM family are co-expressed at the mRNA level with different PI3K pathway genes at varying levels. Genes for this investigation were chosen to depict a wide range of well-studied targets downstream from PIM or PI3K pathways. Spearman’s correlation coefficient was used to determine the strength of the correlation of expression of PIM. PIM1, PIM2 and PIM3 all exhibit positive correlations, indicating they are both upregulated, with Bcl-6. Negative correlations, indicating one gene is downregulated while the other gene is upregulated, were seen between PIM genes and both p85α and mTOR. Overall, it would appear the pathways are predominantly mutually exclusive, particularly downstream, with some notable instances of co-expression.


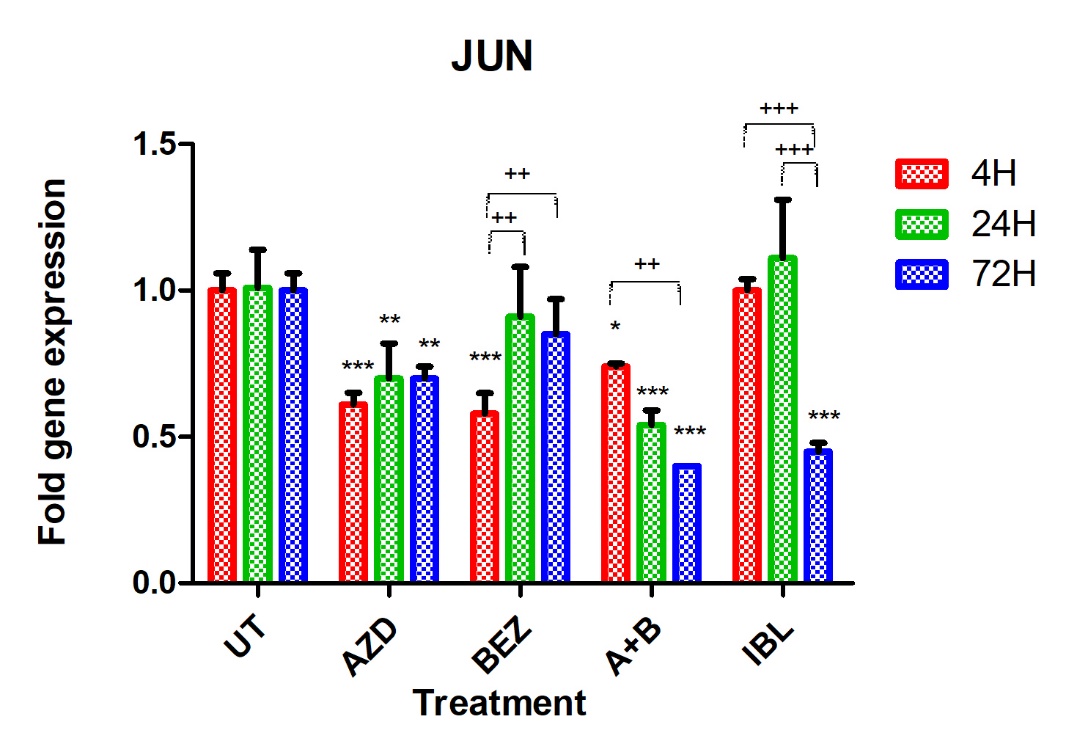


**Figure S3** Fold gene expression of JUN in LNCaP cells demonstrating changes in gene expression at the 4-hour timepoint. For qPCR investigation, LNCaP, C4-2 and C4-2B cells were routinely cultured and treated with AZD-1208, BEZ235, AZD-1208+BEZ235 combined, or AUM302 (here annotated as IBL-302) for 4h, 24h and 72h. Represented as a mean of triplicate values after removal of outliers, with standard deviation as the error bar. All results were normalized to two housekeeper genes, RPLP2 and SDH, and their fold gene expression was calculated using untreated cells as a control. Two-way ANOVA and a Bonferroni post-test were used to determine significance of gene expression changes. (*p<0.05; **p<0.01; ***p<0.001, as compared to UT of the same time point; +p<0.05; ++p<0.01; +++p<0.001, as compared between time points for the same treatment)

Please note: Figure S4 follows, and runs from page 11-23, with its legend following afterwards:
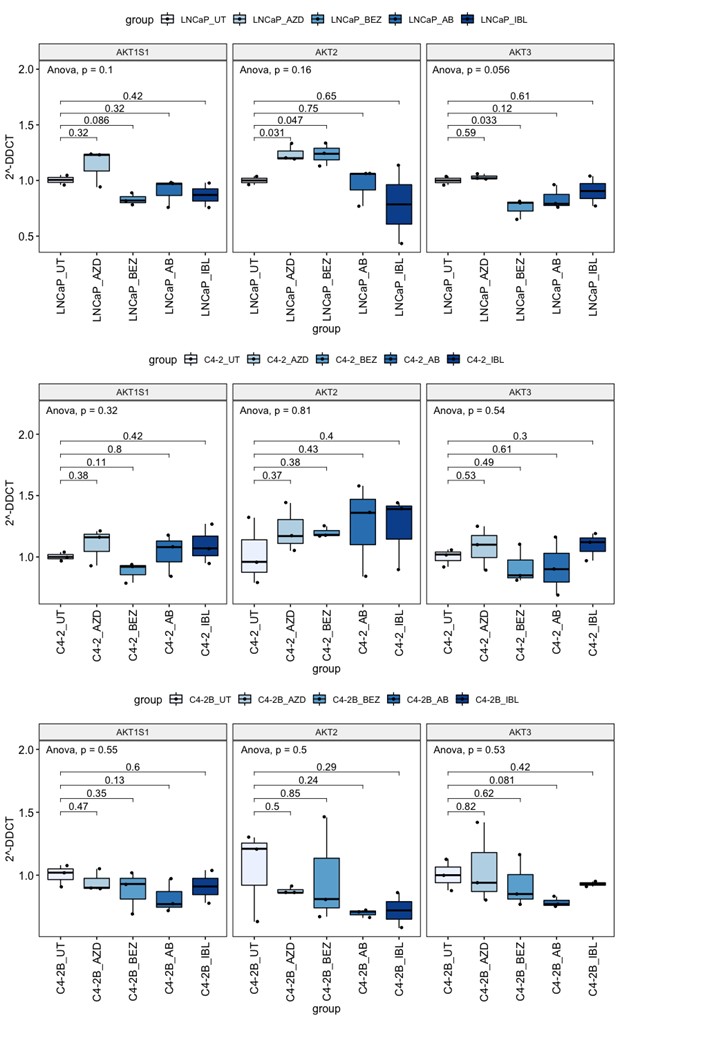

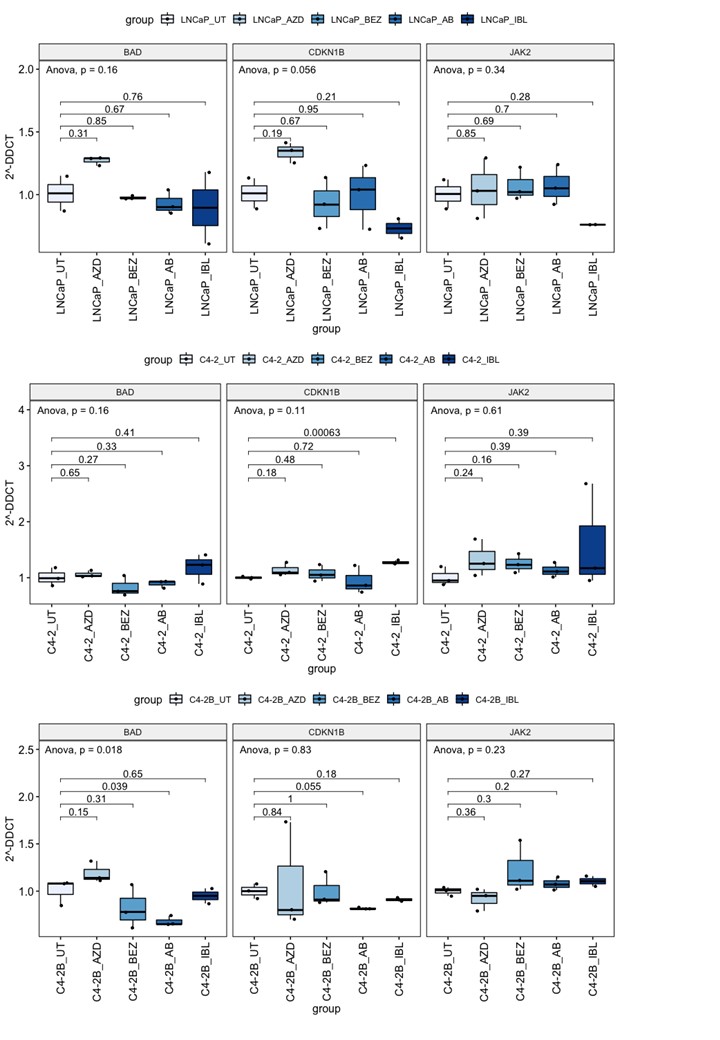

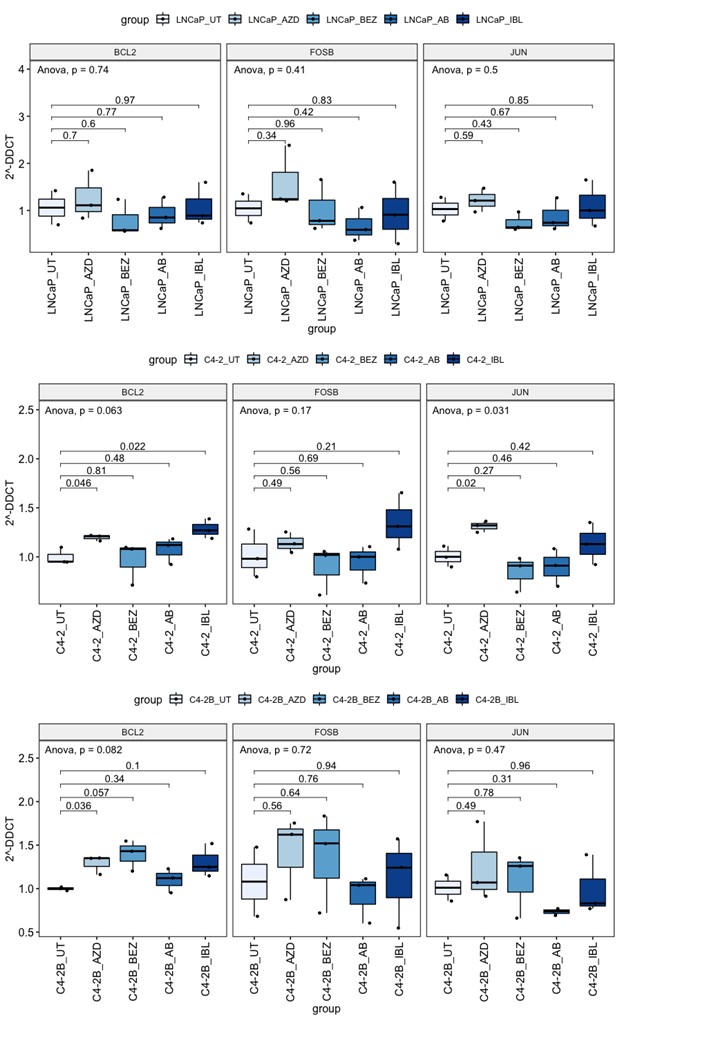

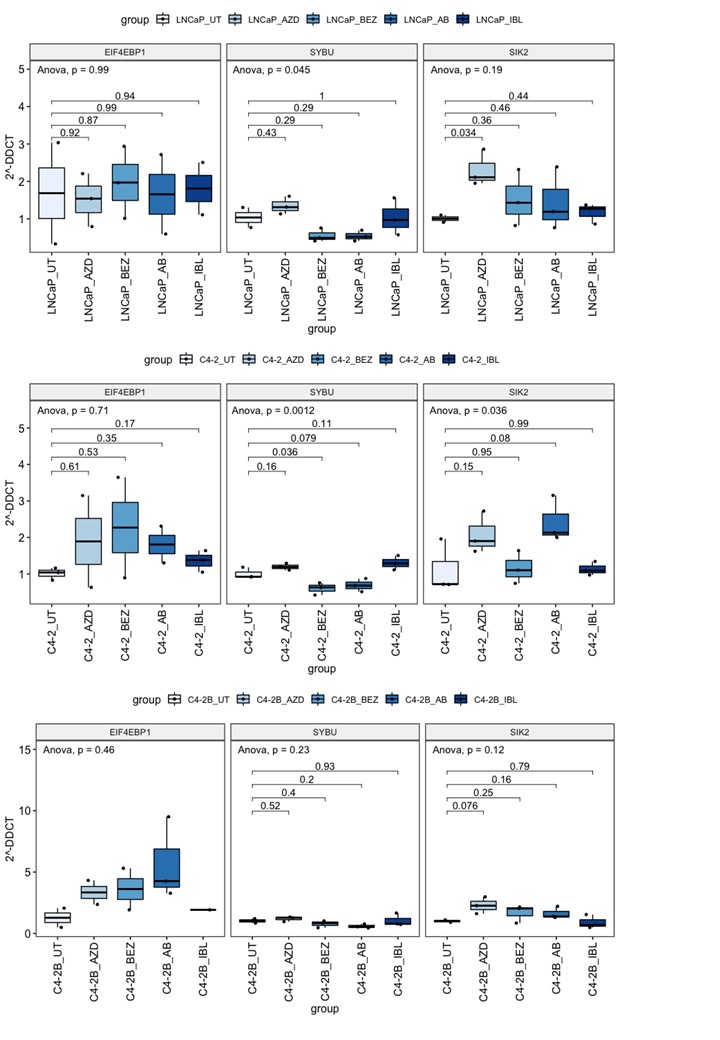

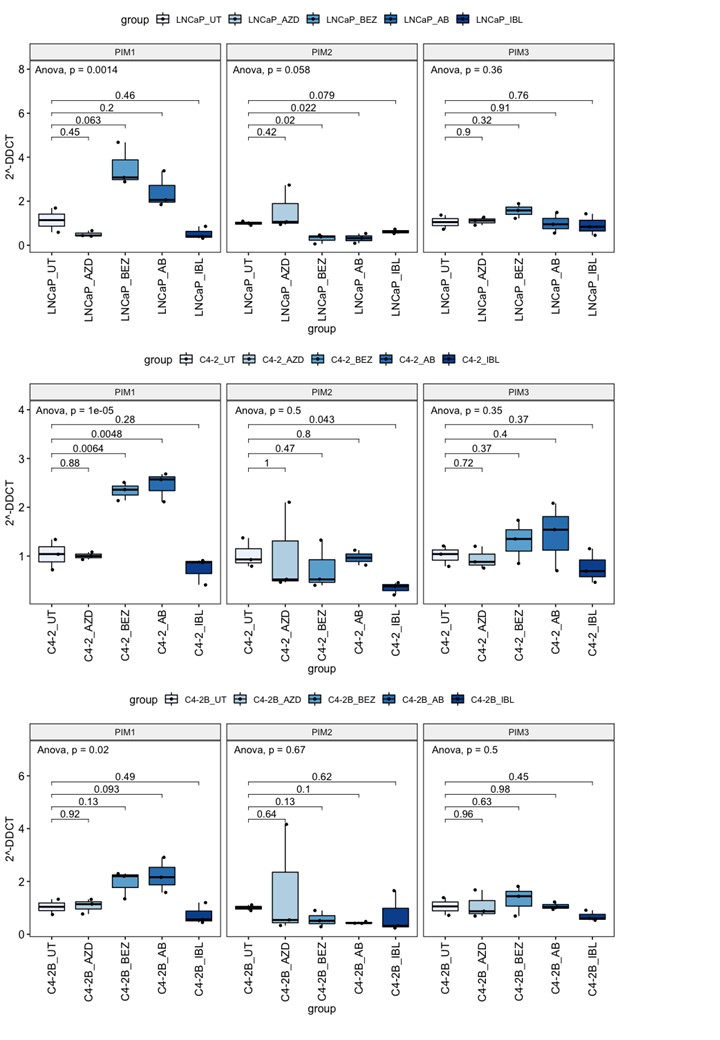

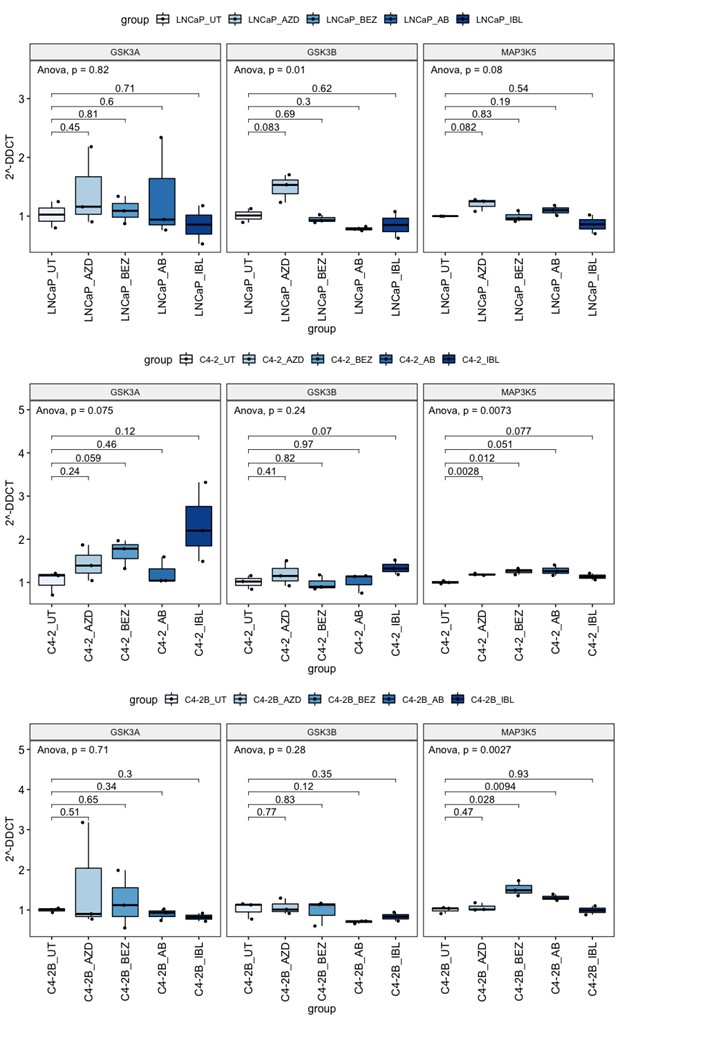

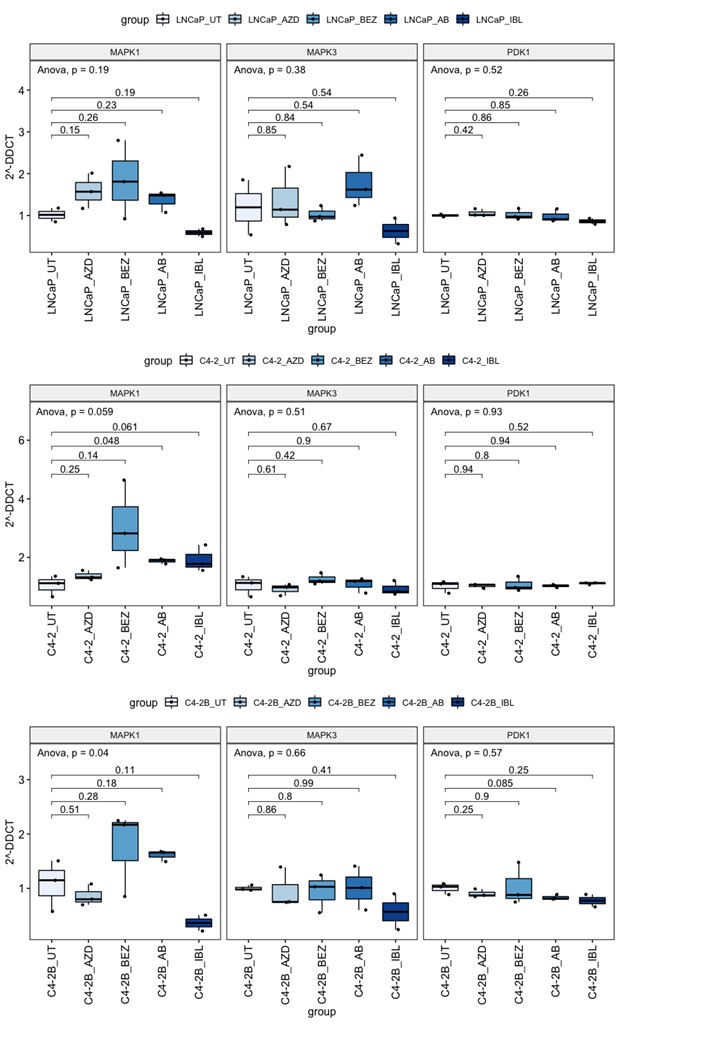

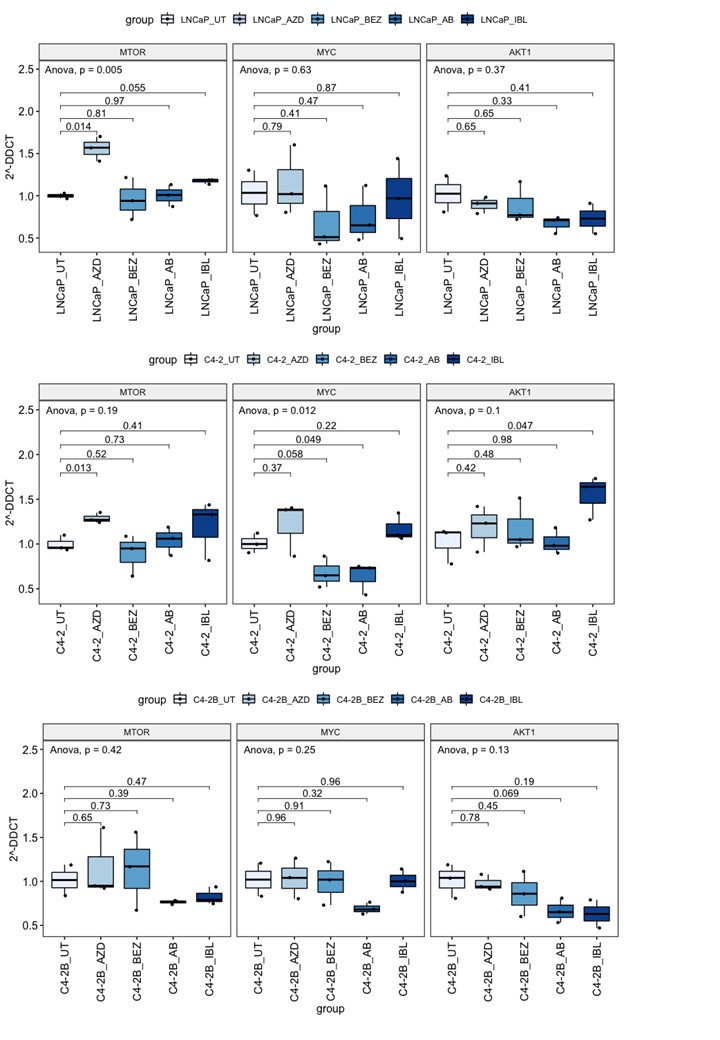

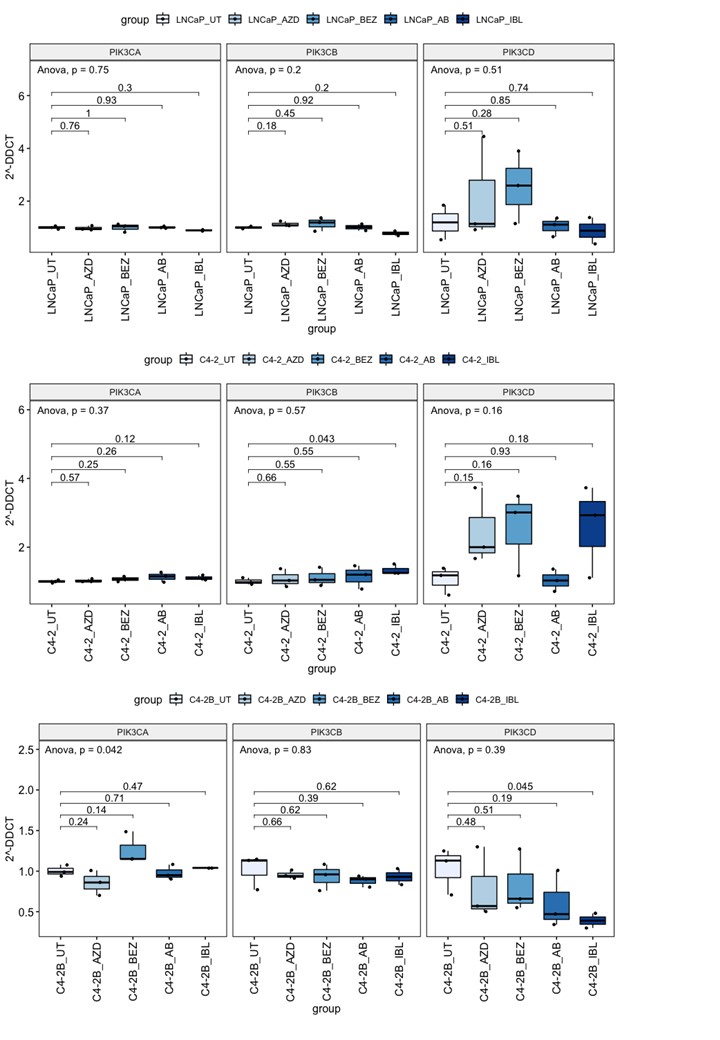

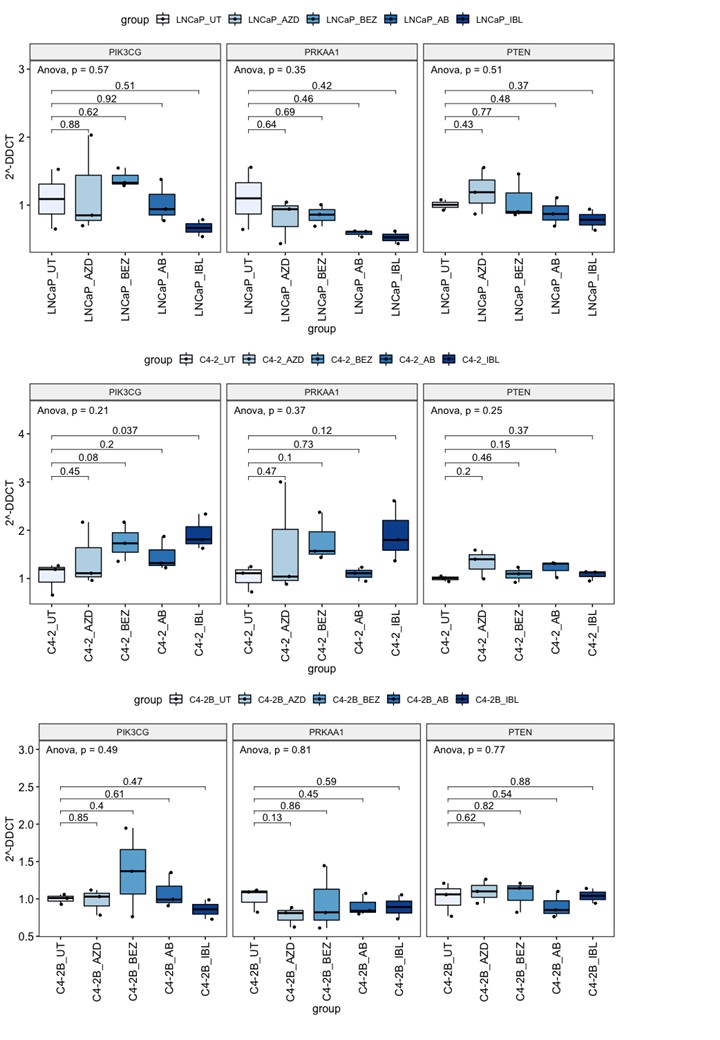

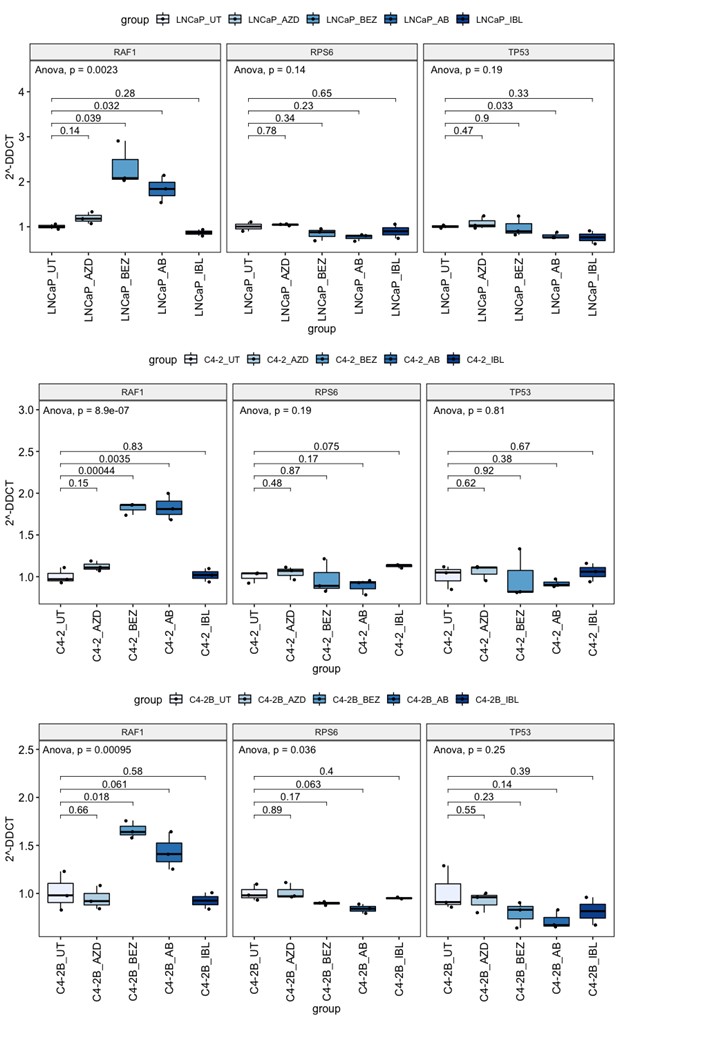

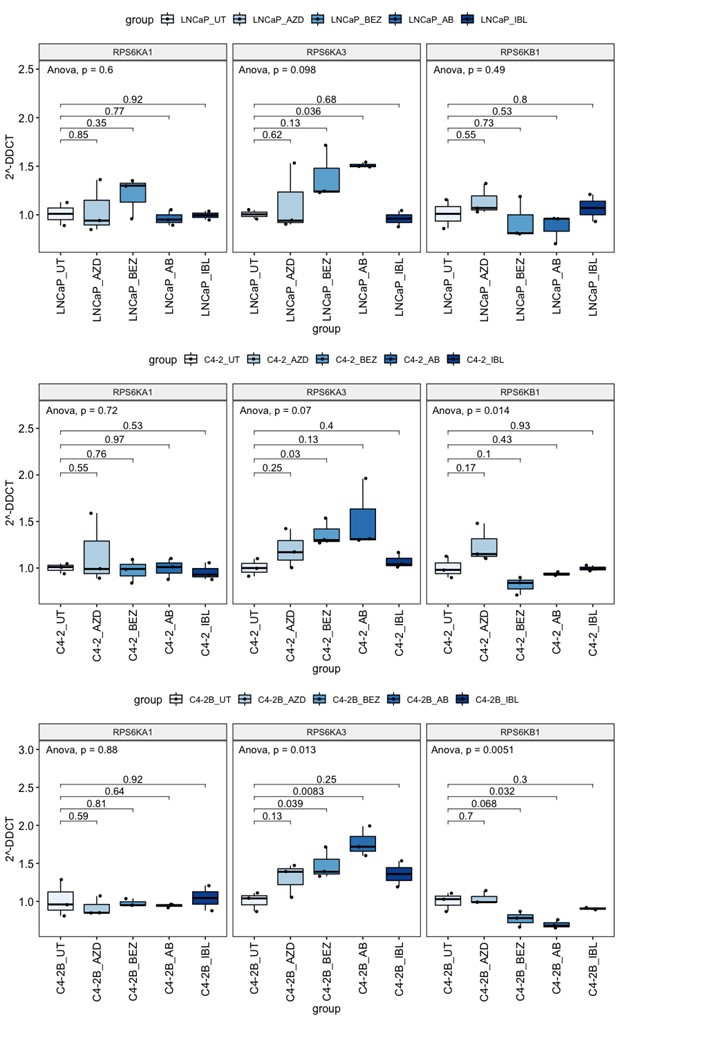

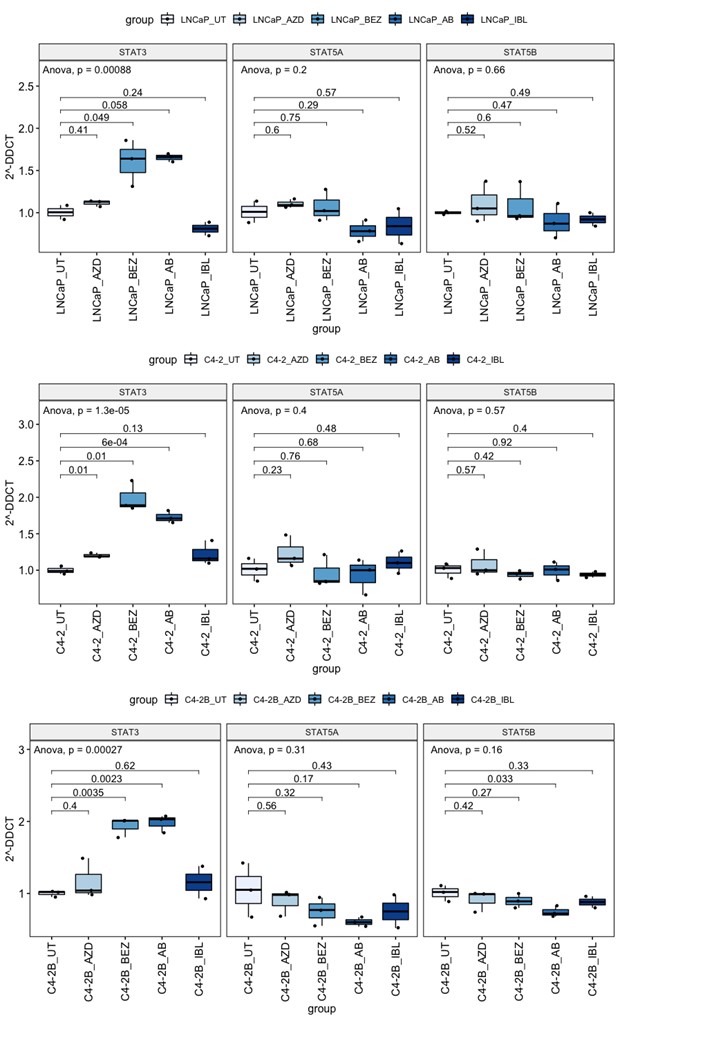


**Figure S4** Co-targeting of PIM and PI3K/mTOR inhibits a wider range of genes than targeting either pathway alone. LNCaP, C4-2 and C4-2B cells were treated for 4 hr with either AZD-1208 or BEZ235 alone, a combination of AZD-1208 and BEZ235, or the multikinase inhibitor AUM302. mRNA was extracted from the resulting samples and used for analysis of gene expression changes using Fluidigm. Results were then analysed using the ΔΔCt method and resulting relative quantification values (2^^^DDCT) were plotted as boxplots reflecting the median and range of the data. One-way ANOVA and a Tukey post-test were used to determine the significance of the findings. Gene included in the gene panel were AKT1S1, AKT2, AKT3, BAD, CDKN1B, JAK2, BCL2, FOSB, JUN, EIF4EBP1, GSK3A, GSK3B, MAP3K5, MAPK1, MAPK3, PDK1, MTOR, MYC, AKT1, PIK3CA, PIK3CB, PIK3CD, PIK3CG, PRKAA1, PTEN, PIM1, PIM2, PIM3, RAF1, RPS6, TP53, RPS6KA1, PRS6KA3, RPS6KB1, STAT3, STAT5A, STAT5B. SYBU and SIK2, shown here, were not included in the final data analysis.

Please note, Figure S5 follows and runs from page 25-27 with the legend following afterwards:
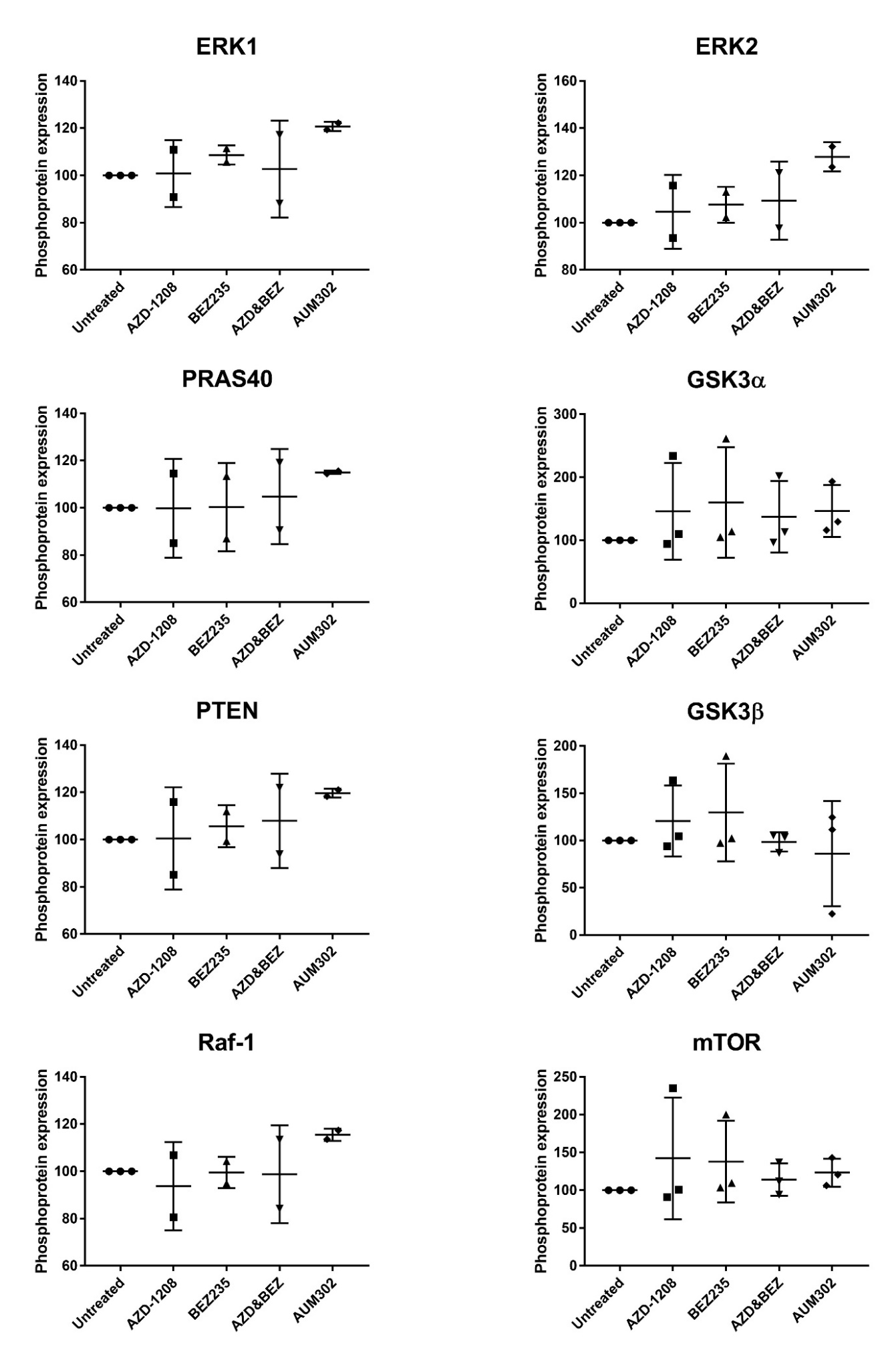

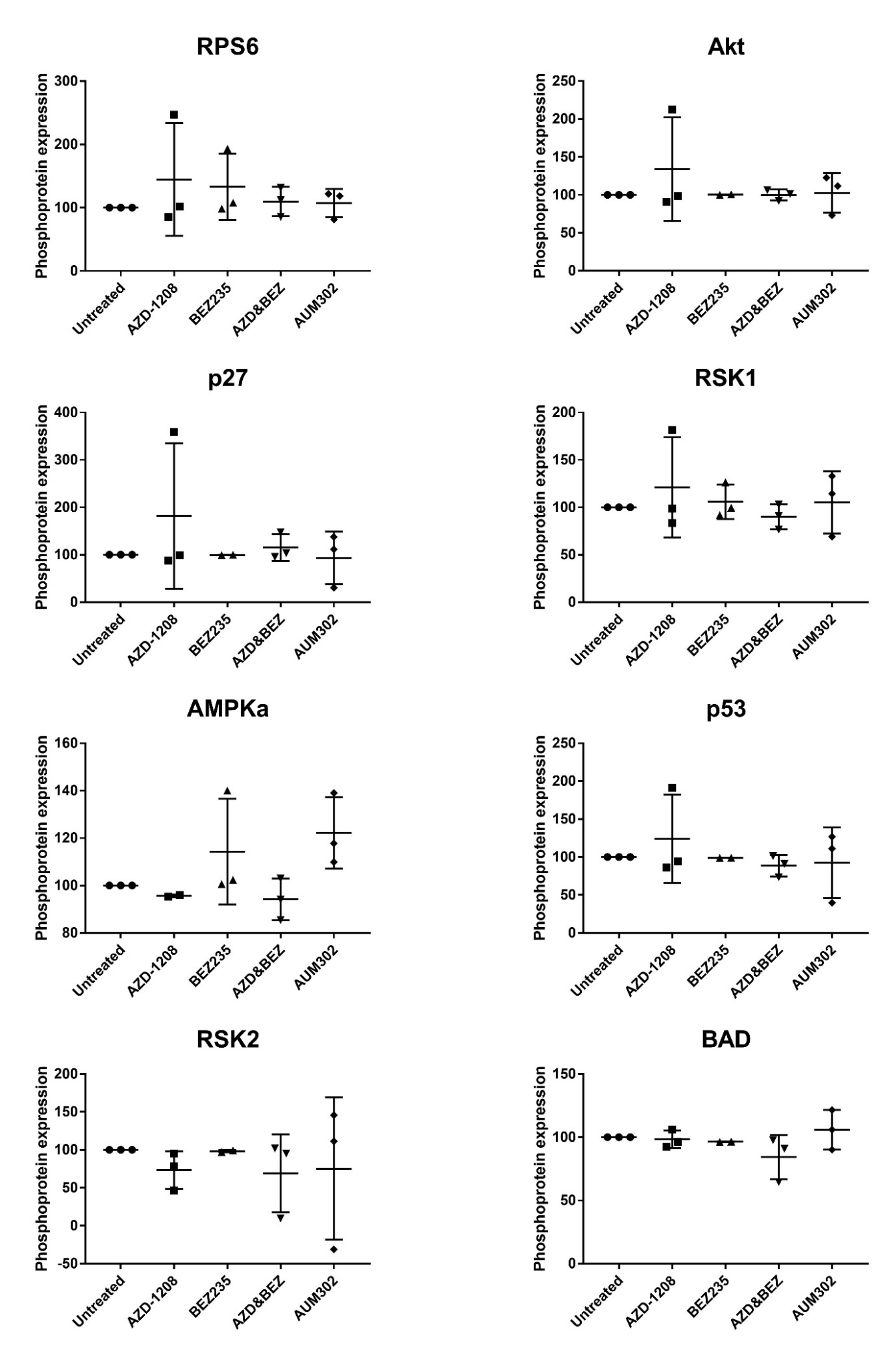

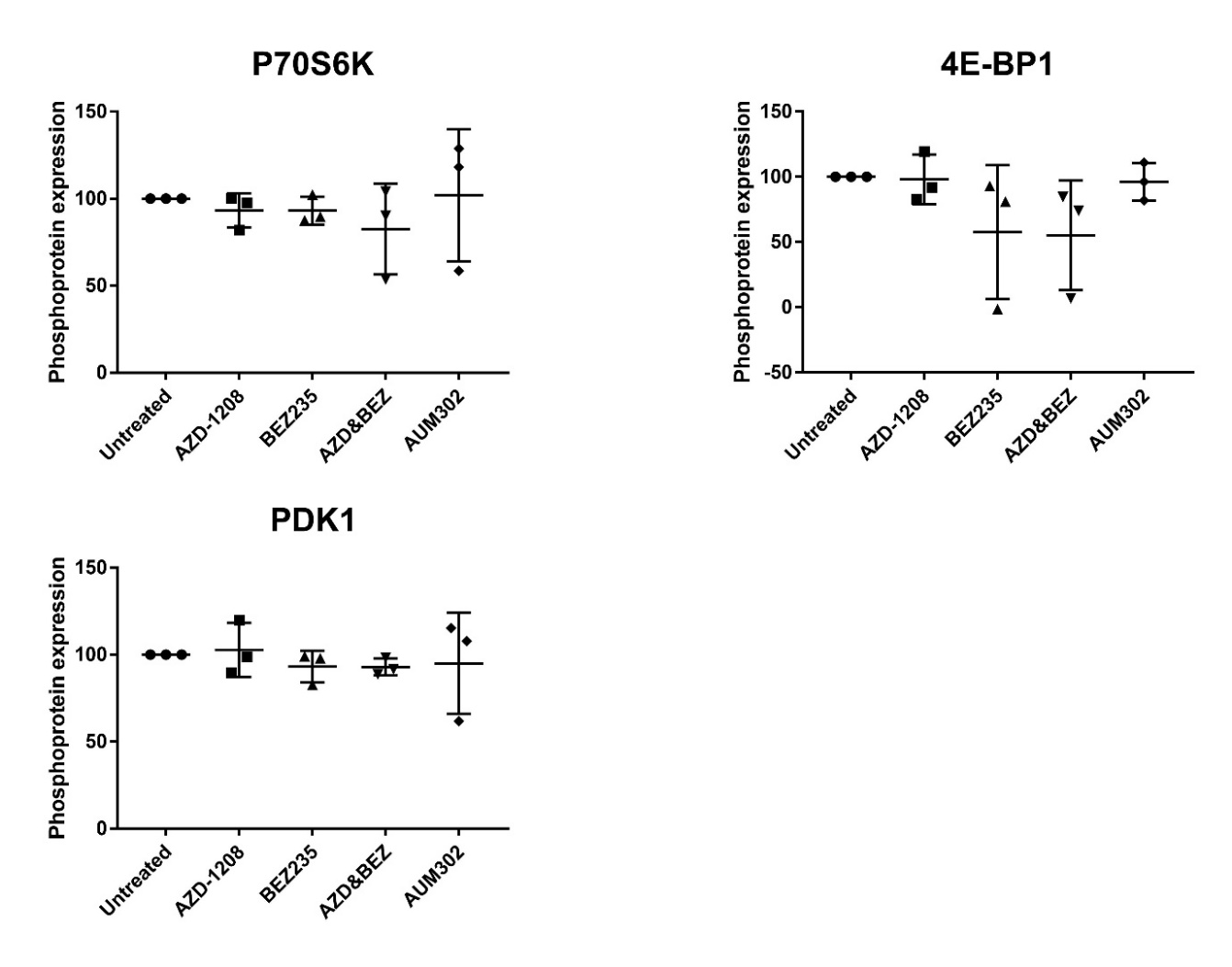


**Figure S5** Combination therapy showed superior inhibition of phosphoprotein levels than any of the monotherapies in LNCaP cells. Cells were cultured and treated for 4 hours with AZD-1208, BEZ235, a combination of AZD-1208 and BEZ235, or AUM302. The proteins were then extracted and quantified using Bradford assay. Changes in phosphoprotein levels were quantified using a phosphokinase array which included key proteins of interest of the PI3K pathway. The results were normalised to positive and negative controls, and then to the untreated control. Whisker plots represent the mean and standard deviation of the data. One-way ANOVA and a Tukey post-test were used to determine the significance of the findings (*p<0.05; **p<0.01; ***p<0.001). None of the datasets for LNCaP cells were significant.


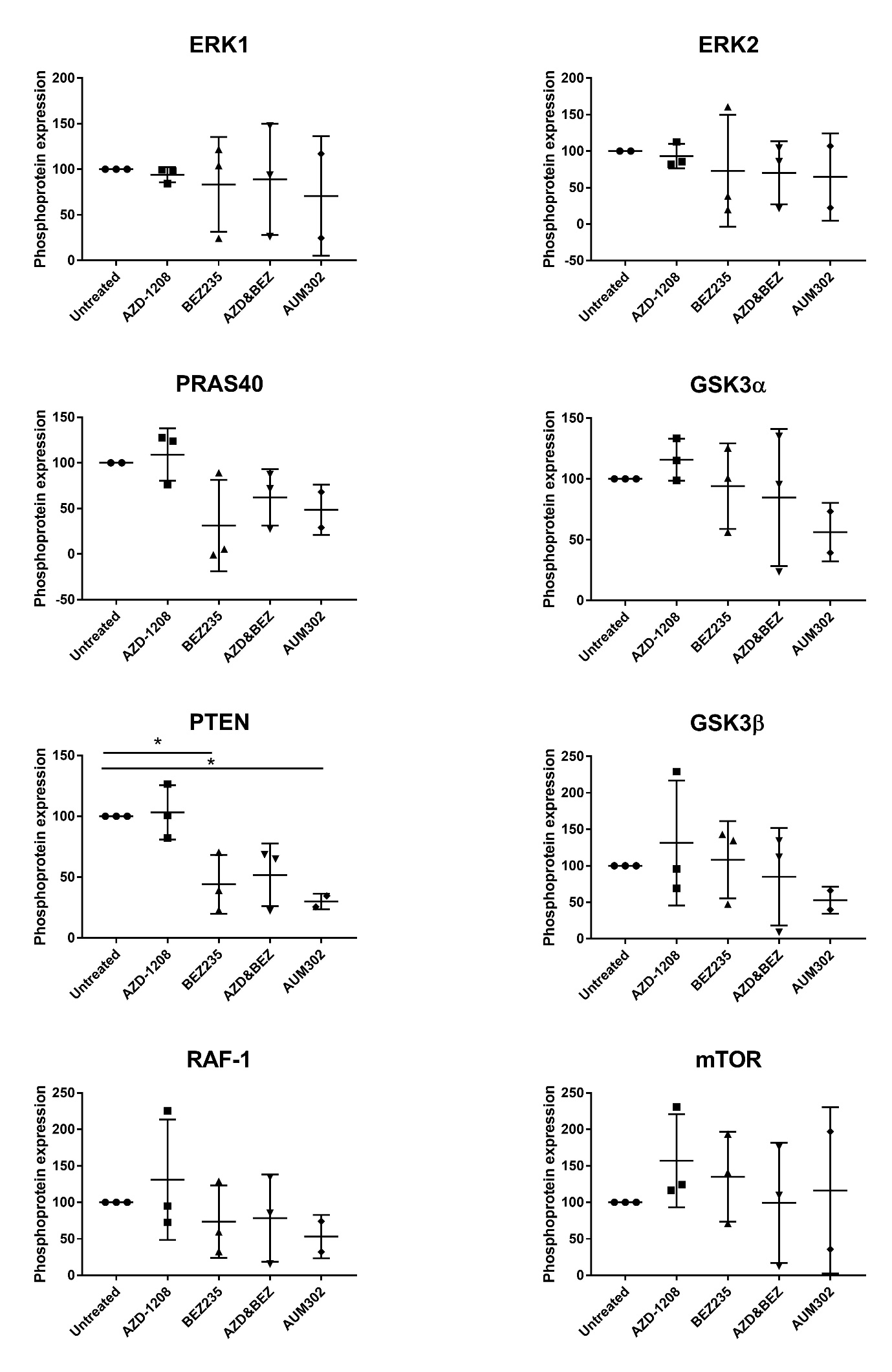

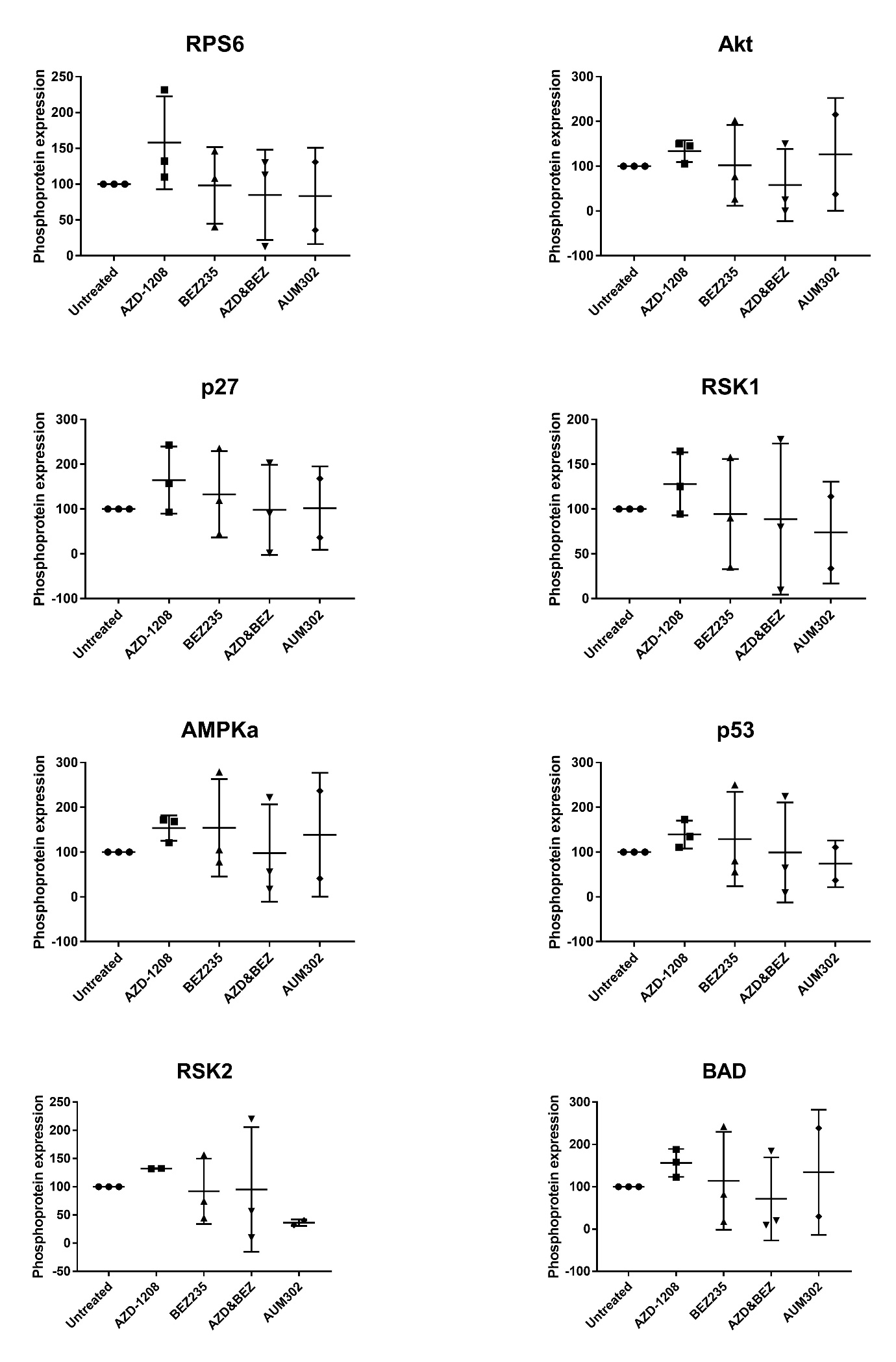

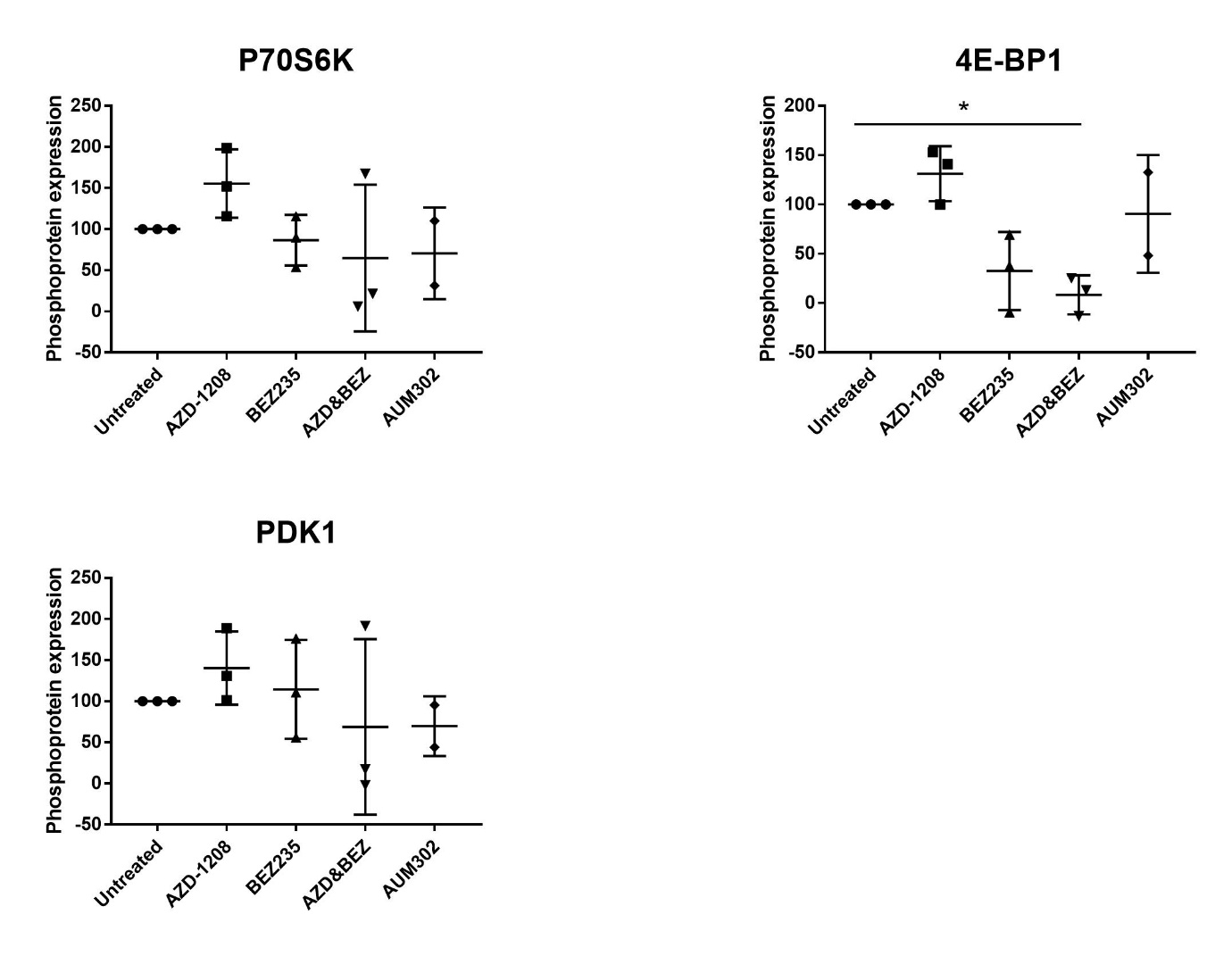


**Figure S6** Combination therapy showed superior inhibition of phosphoprotein levels than any of the monotherapies in C4-2 cells. cells were cultured and treated for 4 hours with AZD-1208, BEZ235, a combination of AZD-1208 and BEZ235, or AUM302. The proteins were then extracted and quantified using Bradford assay. Changes in phosphoprotein levels were quantified using a phosphokinase array which included key proteins of interest of the PI3K pathway. The results were normalised to positive and negative controls, and then to the untreated control. Whisker plots represent the mean and standard deviation of the data. One-way ANOVA and a Tukey post-test were used to determine the significance of the findings (*p<0.05; **p<0.01; ***p<0.001). Data for C4-2 cells was significant only for PTEN and 4E-BP1.


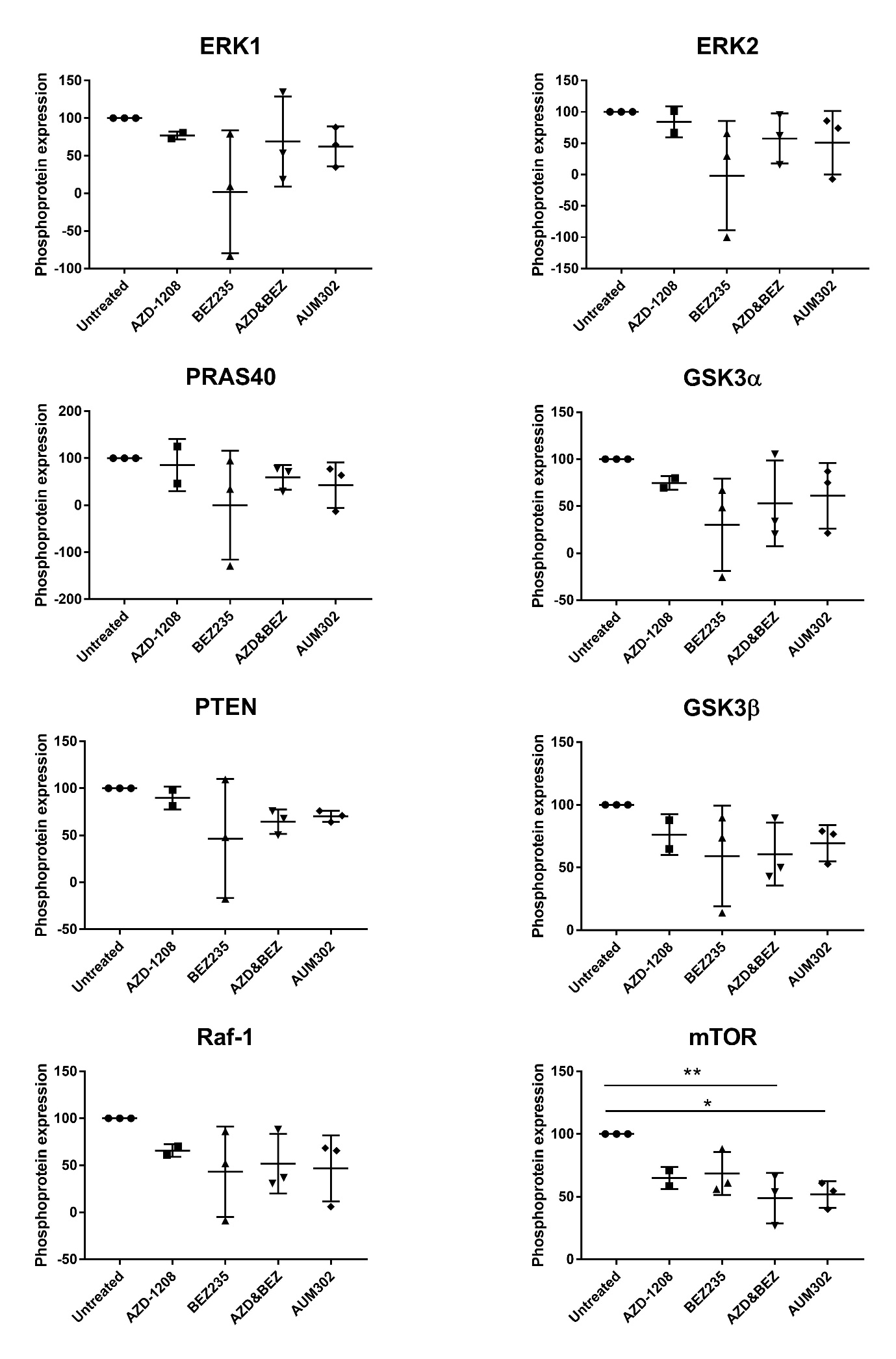

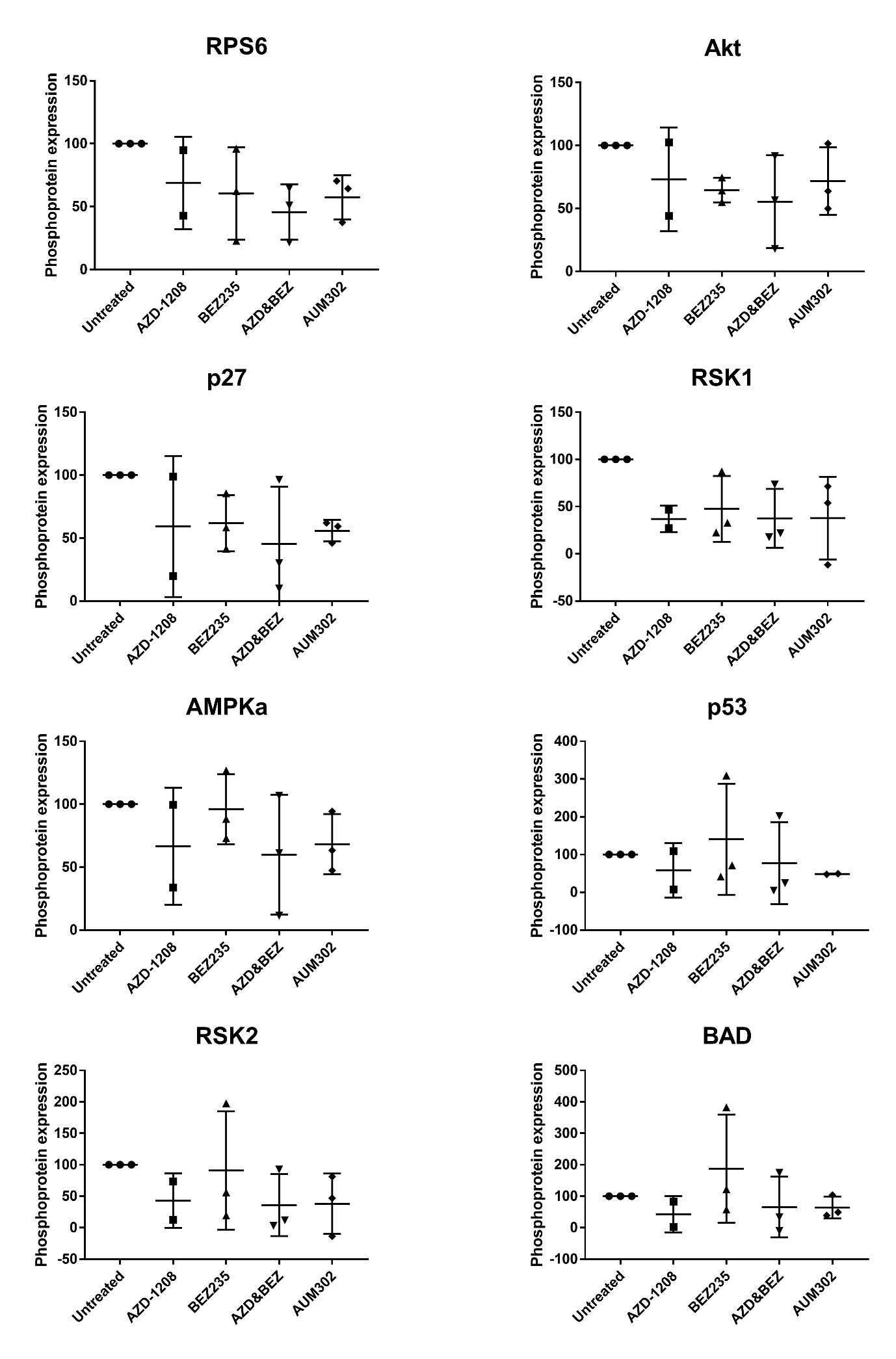

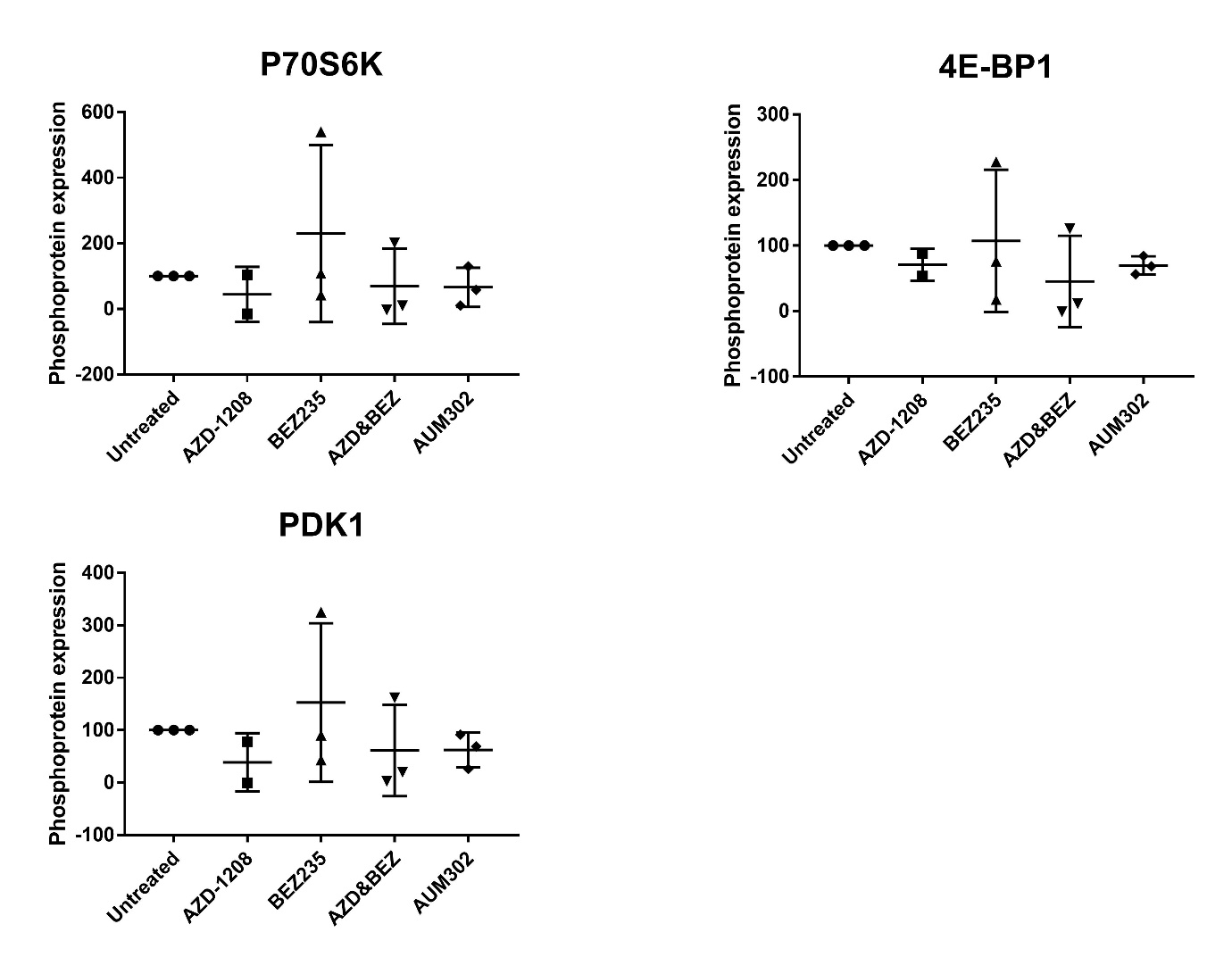


**Figure S7** Combination therapy showed superior inhibition of phosphoprotein levels than any of the monotherapies in C4-2B cells. cells were cultured and treated for 4 hours with AZD-1208, BEZ235, a combination of AZD-1208 and BEZ235, or AUM302. The proteins were then extracted and quantified using Bradford assay. Changes in phosphoprotein levels were quantified using a phosphokinase array which included key proteins of interest of the PI3K pathway. The results were normalised to positive and negative controls, and then to the untreated control. Whisker plots represent the mean and standard deviation of the data. One-way ANOVA and a Tukey post-test were used to determine the significance of the findings (*p<0.05; **p<0.01; ***p<0.001). Data for C4-2B cells was significant only for mTOR.

| Cell line | Total dose of AZD-1208 & BEZ235 [nM] | Fractional affect | Combination Index | CI classification |
| --- | --- | --- | --- | --- |
| LNCaP | 50234.4 | 1.00E-05 | 0.08242 | Synergism |
| C4-2 | 401875 | 1.00E-05 | 0.00844 | Synergism |
|  | 200938 | 1.74E-04 | 0.08957 | Synergism |
|  | 100469 | 1.00E-05 | 0.00211 | Synergism |
|  | 50234.4 | 1.00E-05 | 0.00105 | Synergism |
|  | 25117.2 | 1.00E-05 | 5.27E-04 | Synergism |
| C4-2B | 200938 | 1.00E-05 | 0.96902 | Synergism |
|  | 50234.4 | 1.00E-05 | 0.24225 | Synergism |

**Table S4.** ComboSyn data demonstrating Fractional affect (Fa) and Combination Index, from the Chou-Talalay Theorem (where a CI<1 represents synergistic inhibition, CI = 1 additive effect, and CI >1 antagonism) for LNCaP, C4-2 and C4-2B cells following treatment with increasing concentration of AZD-1208 & BEZ235. Synergism suggests that the combined effect of the drugs used was more potent than either drug alone; additive effect means that the drugs did not potentiate each others’ actions; antagonism suggests an opposing activity between the drugs.


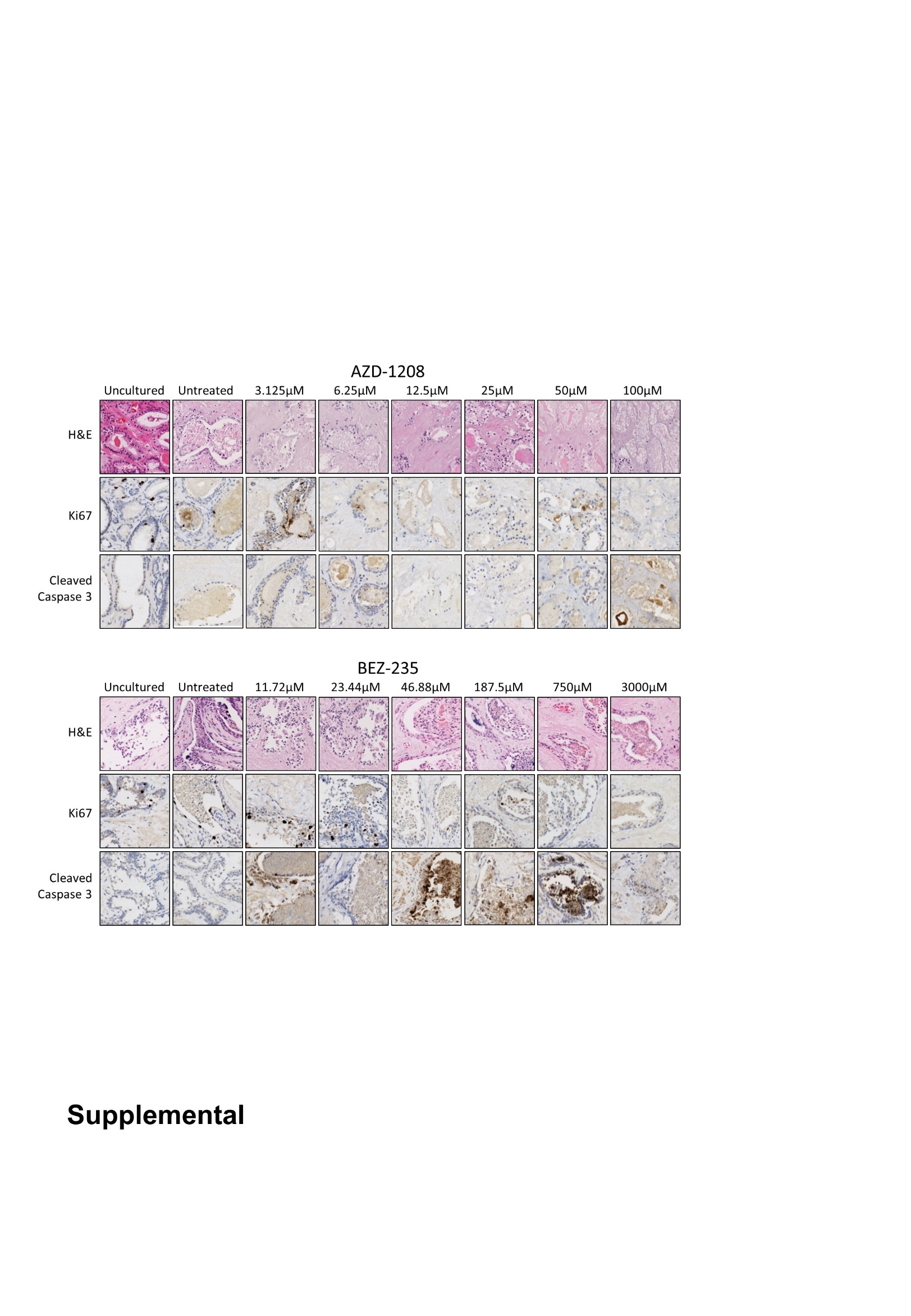


**Figure S8.** Titration of AZD-1208 and BEZ235 in ex vivo prostate tissue culture, followed by H&E, Ki67 and cleaved caspase 3 staining. Prostate tissue was cultured as described previously and underwent treatment with increasing concentrations of AZD-1208 and BEZ235 to determine the effects of the drugs on cell morphology, proliferation and apoptosis, respectively. Brown staining indicates a positive antibody reaction, i.e. higher proliferation or apoptosis. Pink staining is specific for the cell cytoplasm, and blue for the nuclei.

| Treatment | Age | PSA [ng/mL] | Likert | MCCL | Gleason | Grade Group | Staging | Tumour volume [mL] |
| --- | --- | --- | --- | --- | --- | --- | --- | --- |
| AUM302 | 63 | 9.7 | 3 | 14 | 3+4 | 2 | pT2c | 8.1 |
| AZD-1208 | 74 | 5.7 | 5 | 19 | 3+4 | 2 | pT2c+ | 6.5 |
| BEZ235 | 55 | 12 | 3 | 12 | 4+5 | 5 | pT3a | 5.2 |
| AZD-1208 & BEZ235 | 59 | 12.8 | 4 | 28 | 4+3 & tertiary 5 | 3 | pT3b | 6.1 |

**Table S5.** Patient characteristics for the titration of AZD-1208, BEZ235 and AUM302. Prostate cancer samples were obtained from a biopsy, cultured and treated with increasing concentrations of the drugs. Patient age at surgery, serum PSA level (ng/ml) (Prostate Specific Antigen), Likert score, MCLL (Maximum Cancer Core Length), Gleason score, Grade Group, tumour staging and volume are summarized below.
